# Pharmacy stakeholder reports on ethical and logistical considerations in anti-opioid vaccine development

**DOI:** 10.1186/s12910-021-00599-2

**Published:** 2021-03-25

**Authors:** Vincent Wartenweiler, Grace Chung, Amy Stewart, Cody Wenthur

**Affiliations:** 1 School of Pharmacy, University of WI – Madison, 777 Highland Ave, Madison, WI 53705 USA; 2 Department of Pharmacy, UW-Health, 600 Highland Ave, Madison, WI 53792 USA

**Keywords:** Opioid, Drug development, Vaccine, Stakeholder, Ethics, Logistics

## Abstract

**Background:**

As opioid use disorder (OUD) incidence and its associated deaths continue to persist at elevated rates, the development of novel treatment modalities is warranted. Recent strides in this therapeutic area include novel anti-opioid vaccine approaches. This work compares logistical and ethical considerations surrounding currently available interventions for opioid use disorder with an anti-opioid vaccine approach.

**Methods:**

The opinions of student pharmacists and practicing pharmacists assessing knowledge, perceptions, and attitudes toward current and future OUD management strategies were characterized using a staged, multi-modal research approach incorporating a focus group, pilot survey development and refinement, and final survey deployment. Survey responses were assessed using one- and two-way parametric and non-parametric analyses where appropriate, and multi-dimensional matrix profiles were compared using z-tests following an exhaustive combinatorial sum of differences calculation between items within each compared matrix.

**Results:**

Focus group content analysis revealed a high level of agreeableness among participants regarding anti-opioid vaccine technology and a sense of shared ownership regarding solutions to the opioid epidemic at large. Pilot survey results demonstrated subject ability to consider both pragmatic and ethical considerations related to current therapeutics and novel interventions in a single instrument, with high endurance amongst engaged subjects. Access inequality was the most concerning ethical consideration identified for anti-opioid vaccines. Support for anti-opioid vaccine implementation across various clinical scenarios was strongest for voluntary use amongst individuals in recovery, and lowest for mandatory use in at-risk individuals.

**Conclusions:**

Ethical and logistical concerns surrounding anti-opioid vaccines were largely similar to those for current OUD therapeutics overall. Anti-opioid vaccines were endorsed as helpful potential additions to current OUD therapeutic approaches, particularly for voluntary use in the later stages of clinical progression.

**Supplementary Information:**

The online version contains supplementary material available at 10.1186/s12910-021-00599-2.

## Background

An emerging experimental approach to address substance use disorders (SUD) is the development of bioconjugate vaccines that can elicit an immune response directed against small molecules such as opioids [1]. In 2017, the Substance Abuse and Mental Health Services Administration (SAMHSA) found that 1 in 12 American adults (18.7 million) had an SUD diagnosis [2, 3]. Of these, an estimated 2.1 million Americans are affected by an SUD related to opioids, also called an opioid use disorder (OUD) [4]. OUD is defined as the continued use of opioids (prescription or illicit) despite negative drug-related problems [3, 5]. Currently, semi-synthetic and synthetic opioids (other than methadone) are a substantial source of drug overdose deaths—although there was a 4.1% decline in overall drug overdose deaths from 2017 to 2018, the rate of drug overdose deaths involving synthetic opioids increased by 10% over this same period [5, 6]. The current standard of care treatment for OUD consists of medication-assisted treatment (MAT) options augmented with behavioral interventions. Unfortunately, there is an evident gap in care as the number of qualified patients who seek treatment remains low and relapse rates remain disproportionately high. According to the most recently available data, an estimated 18.9 million people needed treatment for a SUD in 2018. However, only 12.6% of this population receive any type of treatment, 19.7% of which was for opioid abuse [7]. However, simply obtaining treatment does not imply a successful recovery. Relapse rates for those patients who receive appropriate addiction treatment are still around 40–60%. Indeed, a comparison study between extended release naltrexone and buprenorphine-naloxone demonstrated 52% and 56% of subjects experienced an opioid-relapse event following treatment with these two interventions, respectively [8]. These high relapse rates for current OUD therapies, coupled with treatment access limitations, and the growing socioeconomic burden of OUD, continue to support interest in the exploration of novel treatments beyond MAT [9–14].

Traditionally, vaccinations were designed prevent widespread infectious diseases; however, increased interest in the use of vaccines for prophylaxis and treatment of substance use disorders have altered the way researchers are thinking about drug development and implementation [15]. Unlike MAT, the general mechanistic principle of vaccine therapy involves the production of anti-drug antibodies which (upon exposure to a drug target) sequester the target molecule in the periphery, thus blunting any rewarding or adverse effects arising from their access to the central nervous system [16]. Preclinical data suggests that anti-opioid vaccines may be used as monotherapy for OUD, or theoretically in combination with the current standard of care [3, 17, 18].

However, despite this favorable data, there are a variety of ethical, logistical, and clinical barriers to consider prior to implementation of anti-opioid vaccines. Pertinent questions regarding public health, society, and individuals have been raised for all vaccines belonging to the evolving class of immunotherapies designed for all types of SUD [19]. Systematic assessment of whether these vaccines will be supported for use in the prevention versus treatment of SUD, and investigations into opinions regarding their appropriate use in specialized populations such as children, military members, incarcerated individuals, and hospitalized patients has been called for by medical ethicists and clinical researchers, but not yet undertaken [20, 21, 26]. Given the enthusiasm for exploration of these dimensions and the paucity of current studies assessing them, this study represents an important first step in analysis of vaccine-based SUD treatments. Furthermore, in addition to the multifaceted ethical dilemmas pertinent to SUD vaccine approaches, logistical barriers surrounding anti-opioid vaccine deployment are likely to intersect with ethical concerns in regard to effective implementation [20]. Such pragmatic considerations are not unlike those seen for other OUD medications, as lack confidence and support, time, and reimbursement have been cited as critical reasons for limited use of buprenorphine-naloxone products [21].

While there are multiple stakeholders who likely have differing positions on these ethical and logistical concerns, front-line practitioners represent an intriguing initial population to survey, as they are in a relatively central position of influence in regard to eventual treatment implementation. Traditionally, the opinions of front-line practitioners and other relevant stakeholders, such as patients, have been delayed until later stages of medication development [22–25]. The near-exclusive reliance on scientific expertise during early phases runs the risk of overweighting this groups’ collective opinion in ethical and logistical areas, where their technical expertise may be irrelevant. Indeed, recent work has found a surprising ability of non-expert opinions to highlight critical ethical considerations related to new technology implementation and has specifically suggested that more intentional alignment could further improve the relevance and efficacy of drug research [26]. While early patient involvement in drug design has been successfully implemented, similar studies addressing analogous considerations from front-line practitioners earlier in drug development are more limited. With these challenges in mind, this research project was undertaken to determine whether the collective clinical knowledgebase of these individuals would meaningfully converge on internally-consistent ethical and logistical positions between established and unfamiliar, experimental therapeutics in early development.

When considering which initial front-line providers to model this early stakeholder engagement process in, pharmacists were selected as this initial study population for multiple reasons. Firstly, pharmacists are uniquely positioned in healthcare to be informational gatekeepers for new drug products, providing pertinent information to prescribers and patients alike [27, 28]. Secondly, pharmacists have a functional working knowledge of current OUD therapeutics, increasingly including the provision of injectable medications for this indication. Finally, pharmacists are frequently tasked with the pragmatic issues related to OUD treatment access, including storage, cost, and coverage of medications. Therefore, a sequence of qualitative focus group analysis, pilot survey development, and final survey deployment to gauge pharmacy student and pharmacist opinions regarding both currently available, and novel vaccine-based treatments for OUD was employed. The primary research aim was to compare the ethical and logistical barriers between current OUD treatments and experimental anti-opioid vaccine therapies, identify situational acceptance of anti-opioid vaccine clinical application in regard to outstanding ethical concerns, and assess the perceived efficacy and utility of these current and experimental interventions for treatment of OUD.

## Methods

### Overall approach

**Fig. 1:**
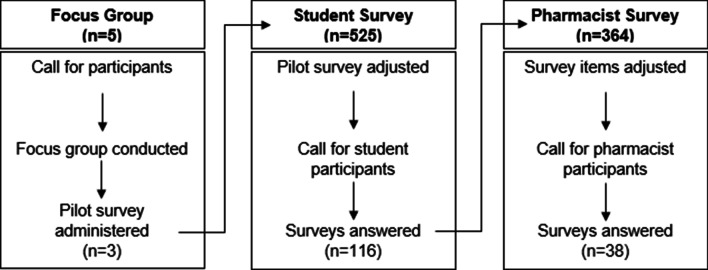
Flowchart describing basic phases of the research project and retention for each phase

Due to the limited scope of literature surrounding practitioner engagement in pre-clinical drug development, the work described here incorporated instrumental refinement across time. An iterative design was employed in order to identify survey domains and times that addressed constructs relevant across broad stakeholder populations. Thus we report a multi-modal, three-phase effort, with the first phase consisting of a focus group and pilot survey for initial item development, the second phase consisting of a student survey for usability and scale analysis, and the third phase consisting of a pharmacist survey for population-specific language refinement and data analysis. Each phase was carried out using best practices (Fig. 1) [29–34]. Information from prior phases was used to inform development and refinement of specific items used in the subsequent phase. For such adaptations, brief cognitive interviewing [35] was used to assess how pilot respondents understood survey items after adjustments were made to the student and pharmacist surveys and after each survey had been distributed. The “think aloud” and probing methods [36] were used to assess homogeneity regarding question interpretation which allowed for comparison across populations. Survey data were captured in REDCap version 7.4.5 [37], exported to Microsoft Excel, and then analyzed in IBM SPSS Statistics version 26, Graphpad Prism version 8 and QSR NVivo version 12.1. Data were coded as integer values in concordance with REDCap response anchors after export to MS Excel. All statistical analyses were undertaken as two-tailed measures, with significance set a priori at α ≤ 0.05. Simulated random results for the sum of difference analysis were generated in Graphpad Prism version 8, using an absolute Gaussian distribution for random scatter of the 59,049 values that were computed.

### Focus group

A digital announcement recruiting focus group participants was posted for PharmD students. Additionally, the School of Pharmacy's Psych and Neuro Special Interest Group executive board was contacted via email to solicit interest for participation. The email described the purpose and requirements of the focus group and asked board members for permission for the study team to recruit members of SIG. Current PharmD, dual PharmD/MPH or PharmD/PhD students were eligible to participate. BS, BA, MS, PhD students and those under 18 years of age were excluded. Upon recruitment, a single 45-min group session was conducted to evaluate knowledge of the experimental domains being investigated. The focus group was facilitated by two investigators who asked participants to respond to open-ended questions regarding the general topics of the survey, and a co-moderator was present to collect participant observation notes. The focus group session was audio recorded, then transcribed onto to the secure data management software REDCap. All personally identifiable information was replaced with non-identifiable titles. Content analysis was performed by two researchers who independently coded the transcription and assigned themes which were then compared for homogeneity.

### Surveys

Using content areas and language developed from the focus group, a pilot survey was developed, which contained Likert scale, rank order, visual-analogue scale, and radio button type questions regarding knowledge of the opioid crisis, use of current pharmacy resources and medication to manage OUD, and perceptions of vaccine-based therapies in pre-clinical development. The pilot survey contained approximately 54 items, requiring roughly 140 physical “clicks”, and an intentional free response question which asked for qualitative feedback on the survey (Supplemental Information). This survey was administered through REDCap, with focus group participants receiving a direct e-mail invitation to participate. The survey was left open for a three-week response period. Directed follow-up efforts were not pursued despite relatively low response rates given that one purpose of this study was to assess cognitive demand and assess the need for development of more directed, shorter instruments. Final surveys were developed from this pilot (see Additional file 1: Supplemental Methods) and delivered to student pharmacists (see Additional file 1: Appendix A; and practicing pharmacists (see Additional file 1: Appendix B).

## Results

### Focus group and pilot survey participation

Five participants were included in the focus group, with at least one participant from each didactic year. Analysis of the transcript indicated that participants were initially slow to respond, and the length of responses were short, but as the session proceeded, participants showed more openness, shared longer responses (including personal stories), and built off one another’s responses to the questions in the guide sheet (See Additional file 1: Appendix C). By minute 11, all participants had spoken at least once and both verbal and non-verbal agreements were witnessed among the group. Seven emerging themes were extracted using the word similarity query in NVivo (see Additional file 1: Figure S1) and identified strong participation amongst all study group members (See Additional file 1: Figure S2) on most topics (See Additional file 1: Figure S3), with direct discussions of opioid overdose having more limited participation (See Additional file 1: Figure S4). Three of five participants in the focus group fully answered the pilot survey, and no comprehension issues were noted in their overall response. The primary feedback on the content of the pilot survey was that there were too many items, and so the final student and practitioner surveys were shortened to accommodate possible mental fatigue, tailoring the included content to address the differential level of expertise and background between the student pharmacist and practitioner surveys.

### Survey sample characteristics

Out of 525 possible respondents, a total of 37 first year students, 53 s year students, and 26 third year students took the student survey (22.1% response). A sample of 364 practicing pharmacists with a Wisconsin state license were invited to complete the practitioner survey, and 38 were taken (10.4% response). The vast majority of student respondents had some experience working in a pharmacy, with 1–2 years of experience being the most common condition, and outpatient sites being the most common work setting (Table 1). Practitioner experience was skewed toward more recent graduates, with less than 5 years in practice being the most frequent condition, and outpatient being the most common practice setting. When considering self-reported practice areas, a small minority of respondents did not associate their practice with any of pain management, addiction therapy, or palliative care, with 44.7% indicating they associated at least one of these with their area of practice (Table 2).

**Table 1:** Respondent characteristics from the student pharmacist survey (n = 116)

Demographics	Total	Percentage
*Over 18 years old*		
Yes	116	100
No	0	0
*Pharmacy school classification*		
DPH-1	37	31.9
DPH-2	53	45.7
DPH-3	26	22.4
*Years working in pharmacy*		
0	7	6.0
Less than 1	11	9.5
1–2	49	42.2
3–4	37	31.9
More than 5	12	10.3
*Work setting*		
Outpatient	75	64.7
Inpatient	26	22.4
Other	8	6.9

**Table 2:** Respondent characteristics from the pharmacist survey (n = 38)

Demographic	Total	Percentage
*Licensed pharmacist*		
Yes	38	100
No	0	0
*Years in practice*		
0–4	18	47.3
5–9	8	21.0
10–14	5	13.2
15–20	2	5.2
More than 20	5	13.2
*Practice setting*		
Outpatient	20	52.6
Inpatient	14	36.8
Other	4	10.5
*Specialty area*		
Pain management	16	42.1
Addiction therapy	7	18.4
Palliative care	11	28.9
None of the above	21	55.3

### Opinions on addressing opioid use disorder

**Fig. 2:**
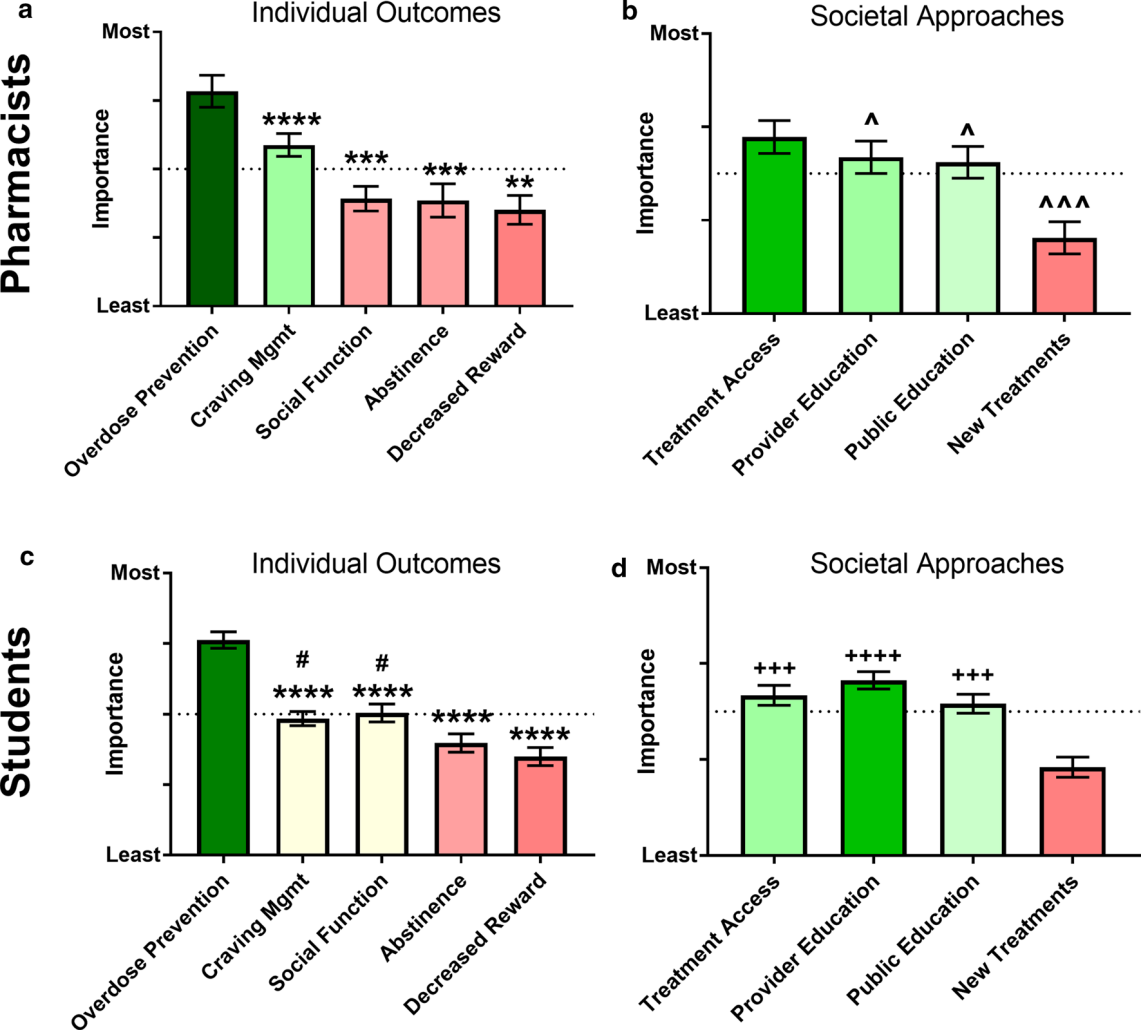
Pharmacist-reported (**a**, **b**) and student-reported (**c**, **d**) rankings for importance of outcomes (**a**, **c**) and approaches (**b**, **d**) with regard to OUD treatment and management. The dotted line on the figures indicates the midpoint of the scale. ANOVA w Tukey’s Correction: **p < 0.01 vs. overdose prevention; ***p < 0.001 vs. overdose prevention; ****p < 0.0001 vs. overdose prevention; ^#^*p* < 0.05 vs. decreased reward; ^^^*p* < 0.05 vs. treatment access; ^^^^^*p* < 0.001 vs. treatment access; ^+++^*p* < 0.001 vs. new treatments; ^++++^*p* < 0.0001 vs. new treatments

To first measure how respondents perceived OUD and its management overall, we assessed their perceptions of interventions at the individual or societal level. When asked to rank order the importance of possible outcomes for individual-level interventions, both students and pharmacists indicated that preventing overdose is significantly more important than other patient-level outcomes (*p* < 0.05 for all comparisons, Fig. 2a–d). Although the overall ranking was quite similar overall, pharmacists prioritized craving management over social function, whereas students ranked these two of nearly equal importance. Both groups ranked craving management as significantly more important than decreasing drug reward, which was considered to be the least important outcome. When ranking approaches taken at the societal level, both students and pharmacists ranked the development of new treatments for OUD as the least important, but pharmacists ranked promoting treatment access as most important, while students ranked educating providers as most important, perhaps reflecting their own present roles.

Students were additionally asked to indicate how severe they perceived the opioid epidemic to be relative to other current healthcare issues. On average, students indicated the opioid epidemic was in the top 30% of national healthcare issues, although variability in this measure covered the full range from 0 to 100% (See Additional file 1: Figure S5). Students were also asked to rank the categories of OUD treatment interventions from most to least important. As a group, students ranked safe medication storage and disposal as significantly less important than any other intervention, with behavioral therapy ranked as the most important overall (*p* < 0.0001, *p* = 0.0048, and *p* = 0.0004 for behavioral therapy, drug use monitoring, and detox vs. safe medication disposal respectively).

### Familiarity with and use of OUD therapeutics

**Fig. 3:**
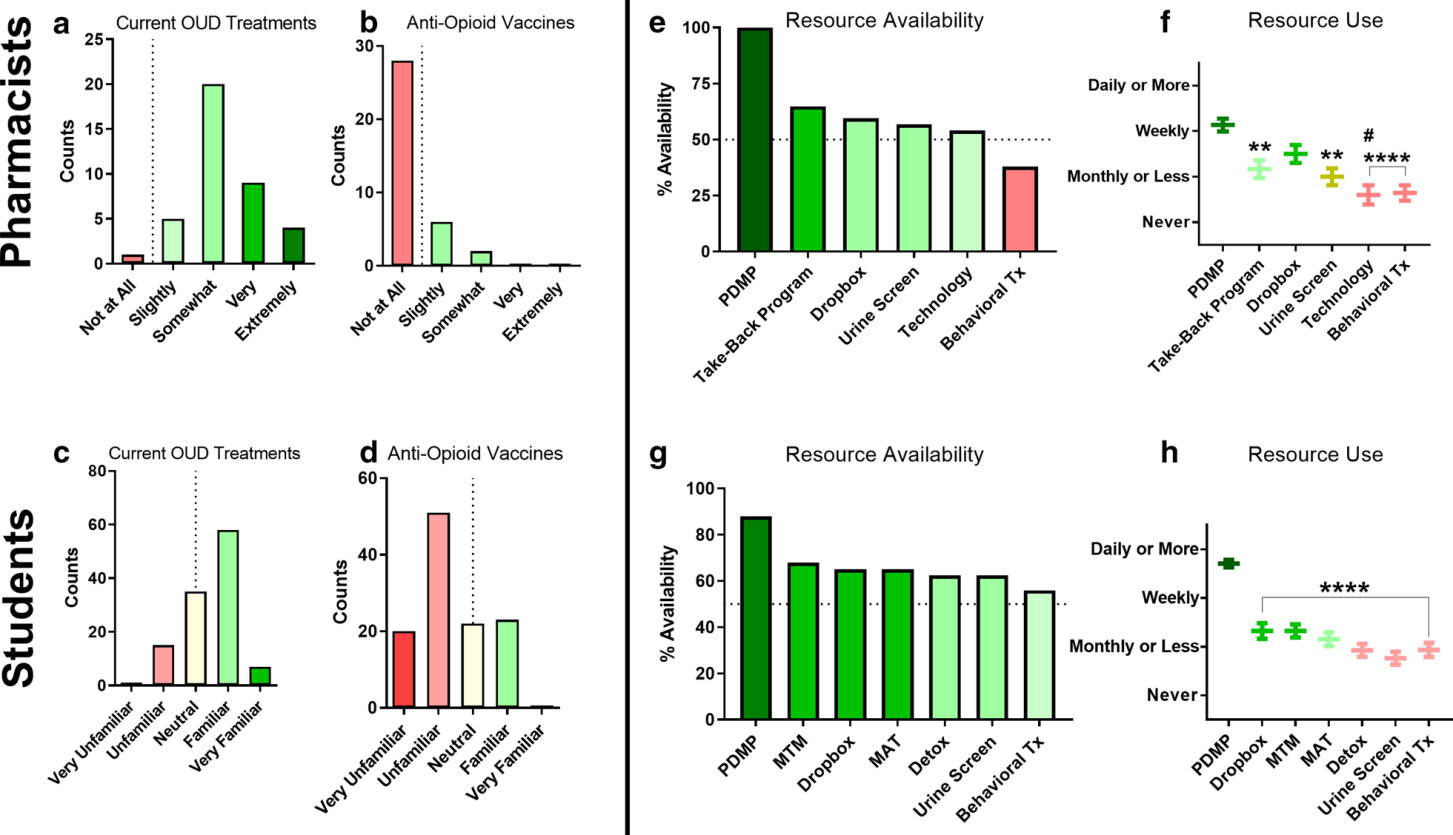
Pharmacist-reported (**a**, **b**) and student-reported (**c**, **d**) familiarity with currently available OUD treatments (**a**, **c**) and anti-opioid vaccines (**b**, **d**). Pharmacist-reported (**e**, **f**) and student-reported (**g**, **h**) availability (**e**, **g**) and utilization (**f**, **h**) of resources for managing OUD. The dotted line indicates the scale midpoint. Data plotted as counts (**a**–**d**), percentages (**e**, **g**) or mean ± SEM (**f**, **h**). Kruskal–Wallis with Dunn’s: **p < 0.01 vs. PDMP; ****p < 0.0001 vs. PDMP; ^#^*p* < 0.05 vs. dropbox

Given that one of the initial criteria for choosing pharmacists as a sample was their high baseline level of knowledge regarding medication interventions, respondents were also asked to assess their knowledge regarding OUD therapeutics and anti-opioid vaccine approaches (Fig. 3a–d). Both students and pharmacists indicated a greater degree of familiarity with current treatment options when compared to anti-opioid vaccines. This difference was apparent when using a bipolar scale for the student survey, but adjustment to a unipolar scale for the practitioner survey further highlighted the large number of individuals with no prior knowledge of anti-opioid vaccine approaches whatsoever. Stratification of student data by year in school and pharmacist data by years in practice revealed students’ confidence in knowledge of current therapeutics increased with years of training (*p* = 0.03). This significance was not reflected in the pharmacist population. Additionally, there was no equivalent trend regarding familiarity with VBTs in either students or pharmacists.

In order to more closely measure functional familiarity with current resources and OUD medications, and additionally to provide a baseline analysis for which types of resources were most likely to penetrate into a pharmacy practice environment, respondents were further asked about their use frequency for other potential interventions, including non-pharmacologic interventions (Fig. 3e–h). Compared to all other available resources, the prescription drug monitoring program (PDMP; i.e. controlled substance tracking database) was both the most available resource and was reported as significantly more frequently utilized by pharmacists than all other resources (*p* < 0.05 for all comparisons) and significantly more utilized at students’ places of work (*p* < 0.0001 for all comparisons). For the examined resources, availability was closely matched with frequency of use overall, with the notable exception that dropboxes appear more frequently used than take-back programs, despite being less popular.

**Fig. 4:**
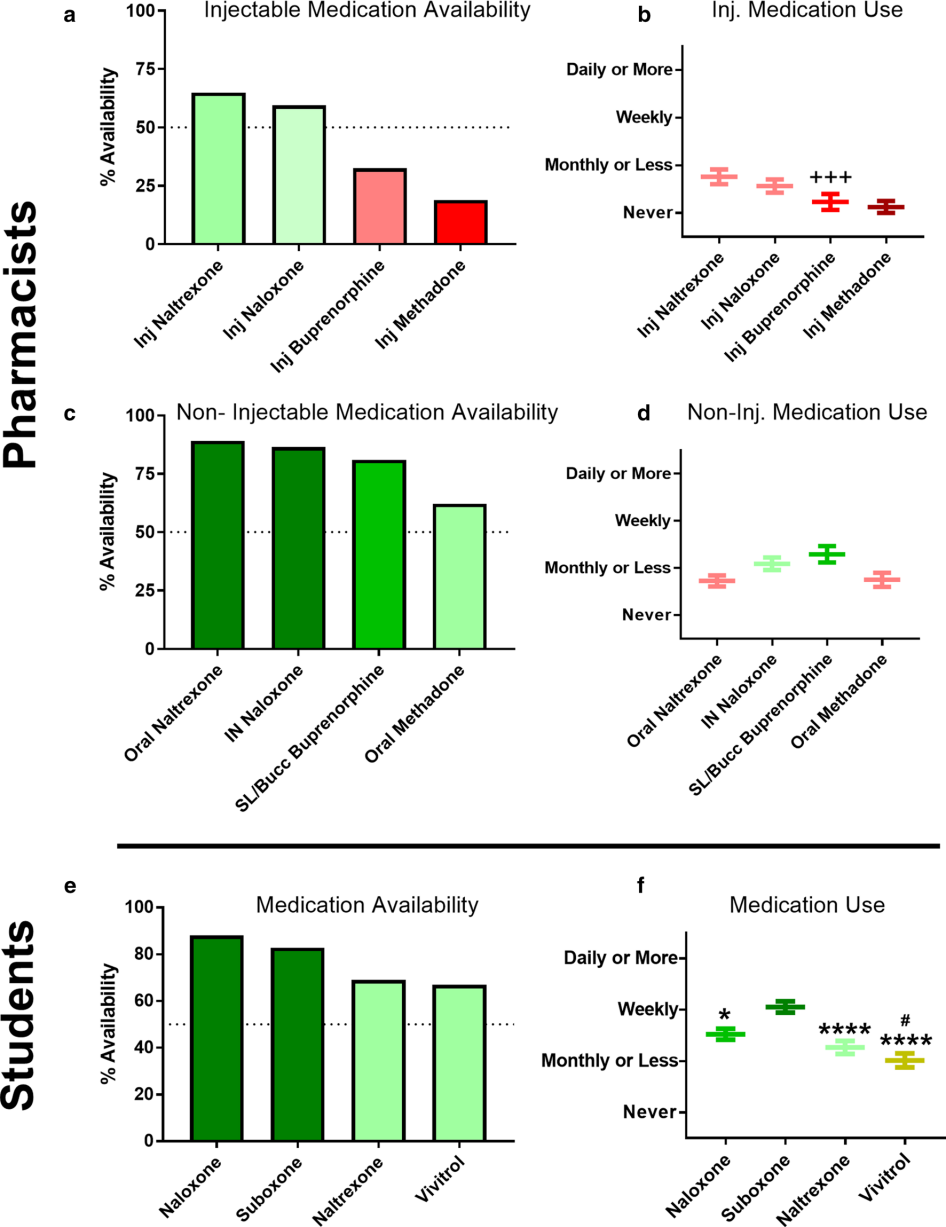
Pharmacist-reported availability (**a**, **c**) and utilization (**b**, **d**) of injectable (**a**, **b**) and non-injectable (**c**, **d**) medication formulations for managing OUD. Student-reported availability (**e**) and utilization (**f**) of medications used to manage OUD. The dotted line (**a**, **c**, **e**) indicates the scale midpoint. Data plotted as percentages (**a**, **c**, **e**) or mean ± SEM (**b**, **d**, **f**). Kruskal–Wallis with Dunn’s: ^+++^*p* < 0.001 vs. SL/buccal buprenorphine; *p < 0.05 vs. Suboxone®; ****p < 0.0001 vs. Suboxone®; ^#^*p* < 0.05 vs. Naloxone

With this information regarding non-medication resource use in hand, we further assessed respondents’ perceptions regarding availability and utilization of specific medications for management of OUD and its associated risks (Fig. 4a–f). Overall, naloxone and buprenorphine-naloxone were available at roughly 80% of pharmacies where students worked and were used weekly or more. Naltrexone (oral tablets) and Vivitrol® were available at roughly 60% of these pharmacies and were used about monthly or less. For the practitioner survey, respondents were specifically asked to assess the availability and utilization of different dosage forms of these medications, as a means to identify whether an injectable dosage form seemed to be a structural barrier to use overall. Injectable forms of naltrexone and naloxone were available at just over 50% of pharmacies while injectable buprenorphine, and methadone were available at about 25% or less of pharmacies. Conversely, oral naltrexone and intranasal naloxone were available at 89% and 86% of pharmacies respectively while sublingual/buccal buprenorphine and oral methadone were available at 81% and 62% of pharmacies. Utilization of injectable dosage forms was lower overall, aside from IM naltrexone, but was significantly lower for only buprenorphine (*p* = 0.0003). In contrast to the results with the resources, availability of medications was not as closely associated with frequency of use, particularly for the oral medication formulations.

### Efficacy of current interventions and perceptions of anti-opioid vaccine utility

**Fig. 5:**
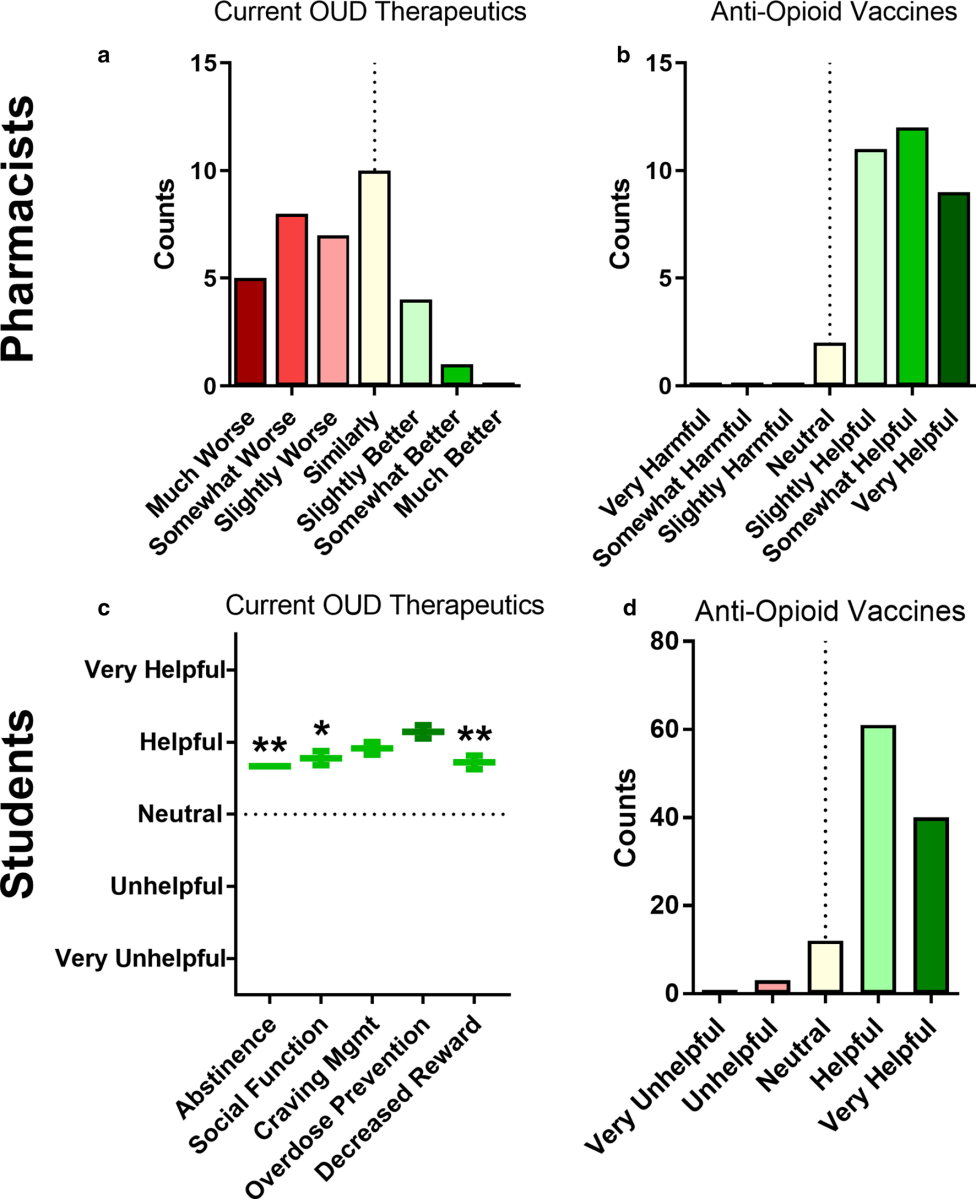
Pharmacist-reported perceptions of current OUD treatment efficacy as compared to other medication-managed therapeutic areas (**a**) and perceived impact of anti-opioid vaccines for OUD treatment (**b**). Student-reported perceptions of current interventions’ effectiveness in promoting individual outcomes (**c**) and perceived impact of anti-opioid vaccines for OUD treatment (**d**). The dotted lines indicate a neutral response. Data plotted as counts (**a**, **b**, **d**) or mean ± SEM (**c**). Kruskal–Wallis with Dunn’s: *p < 0.05 vs. overdose prevention; **p < 0.005 vs. overdose prevention

The overall perceived utility of current OUD therapeutics and anti-opioid vaccines was then assessed (Fig. 5a–d). When students were asked to consider how helpful or unhelpful currently available interventions were for meeting patient-specific outcomes (abstinence, social functioning, craving management, overdose prevention, and decreased drug reward) there was general agreement that current treatments are helpful for meeting these outcomes overall, using a bipolar scale. After being asked how helpful or unhelpful they believed that anti-opioid vaccines will be as an addition to current OUD therapies, the majority of students also indicated vaccines would likely be either helpful or very helpful. Given the limited difference observed between efficacy in treating specific outcomes, and the overall bias toward helpfulness that was observed when using the bipolar scale with students, the question addressing utility of current OUD treatments was amended when asked to pharmacists. Specifically, it was adjusted to get additional information regarding the relative helpfulness of OUD treatments as compared to available treatments for other conditions. When asked the question using this approach, about one-third of pharmacists believed current treatments are slightly worse or much worse than treatments available for other therapeutic areas. Considering the difficulties of predicting relative efficacy for available versus hypothetical treatments, pharmacists were still presented with a bipolar question regarding their perception of how helpful or harmful anti-opioid vaccines would be for treatment of OUD. The majority of respondents indicated that vaccines would be slightly, somewhat, or very helpful for OUD treatment outcomes.

### Perceived logistical barriers and ethical concerns with current OUD treatments and anti-opioid vaccines

**Fig. 6:**
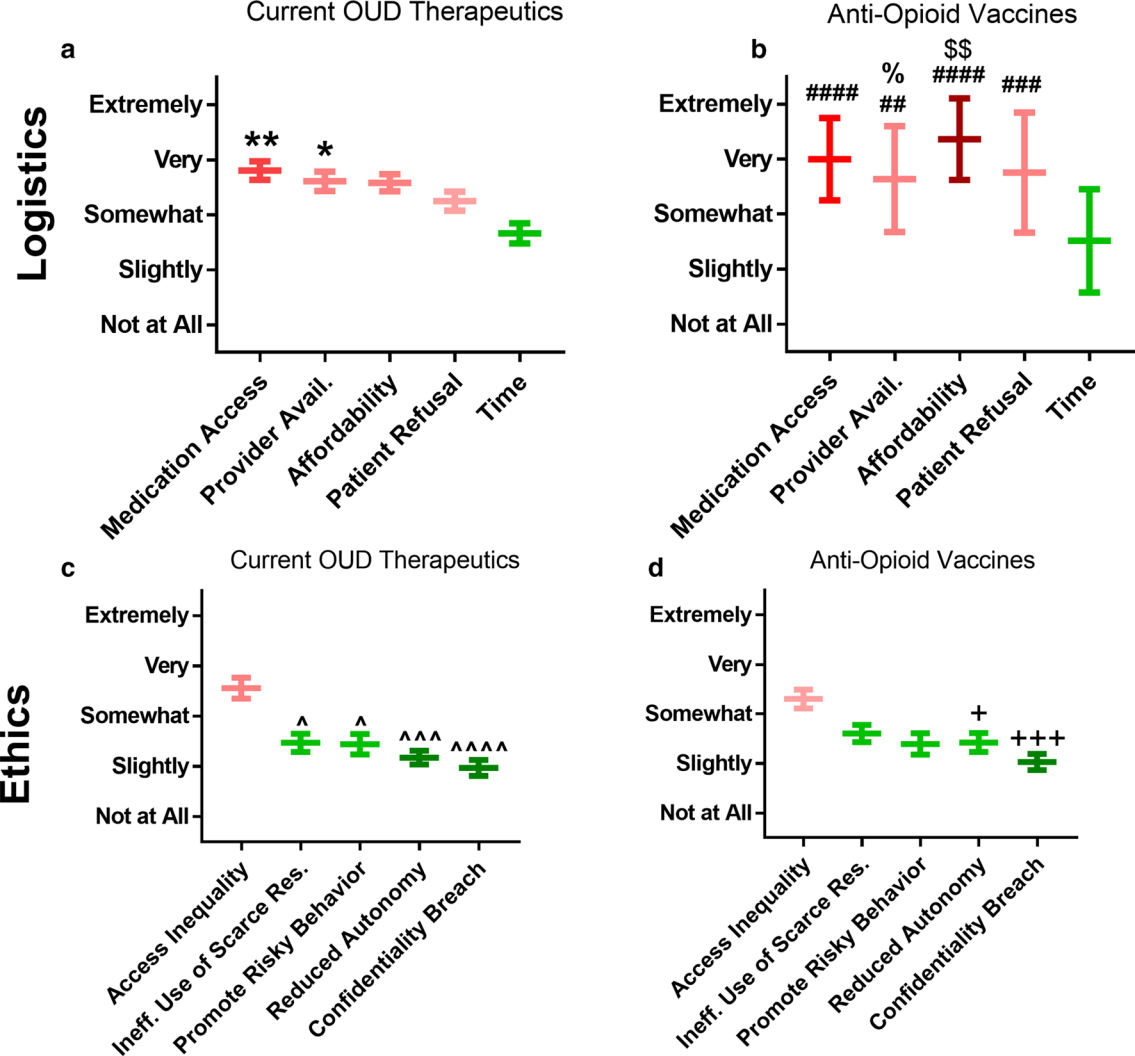
Pharmacist-reported perceptions regarding logistical barriers (**a**, **b**) and ethical concerns (**c**, **d**) for current treatments (**a**, **c**) and anti-opioid vaccines (**b**, **d**). All data plotted as mean ± SEM. Friedman with Dunn’s: *p < 0.05 vs. time; **p < 0.01 vs. time; ^##^*p* < 0.005 vs. time; ^###^*p* < 0.001 vs. time; ^####^*p* < 0.0001 vs. time; ^%^*p* < 0.05 vs. provider availability; ^$$^*p* < 0.01 vs. affordability of current OUD Treatments ^^^*p* < 0.05 vs. access inequality; ^^^^^*p* < 0.001 vs. access inequality; ^^^^^^*p* < 0.0001 vs. access inequality; ^+^*p* < 0.05 vs. access inequality; ^+++^*p* < 0.0005 vs. access inequality

When asked about logistical barriers to both current OUD treatments and anti-opioid vaccines, student responses did not differentiate greatly between these two classes. In terms of the logistical and ethical barriers considered, all of them were considered significant barriers or concerns, although variability was greater within those responses that concerned anti-opioid vaccines (See Additional file 1: Figure S6). This observed bias toward one half of the bipolar scale again prompted the use of a modified, unipolar scale when administering the analogous questions on the pharmacist survey. In order to improve quantification of differences, exact matching of items for current OUD therapeutics and anti-opioid vaccines was introduced as well. Using this modified scale and approach, pharmacists indicated time was the least worrisome logistical barrier, as compared to medication access, provider availability, affordability, and patient refusal for the vaccine-based therapies (*p* < 0.05 for all comparisons) and medication access or provider availability for current therapies (*p* < 0.05 for both comparisons) (Fig. 6a, b). Affordability and patient refusal were noted as more serious barriers for anti-opioid vaccines than for current therapeutics, while the magnitude of concern for the other barriers was similar on average. A larger degree of variability was again seen for the anti-opioid vaccine items. When assessing ethical considerations, pharmacists also ranked access inequality as a very important ethical concern for current and vaccine-based treatments (Fig. 6c, d). For current therapies, access inequality was ranked as a significantly more important treatment barrier than any of inefficient use of scarce resources, promotion of risky behavior, reduced patient autonomy, and potential confidentiality breaches (*p* < 0.05 for all comparisons). For vaccine-based therapies, access inequality was also ranked as a significantly more important barrier than both reduced autonomy (*p* = 0.03) and confidentiality breaches (*p* = 0.0004). Reduced autonomy was rated as more concerning for anti-opioid vaccines than current therapeutics, while the other items were rated similarly. Amongst logistical and ethical considerations tested pairwise, only affordability was found to be significantly different with respect to current therapeutics versus anti-opioid vaccines (*p* = 0.0085).

**Fig. 7:**
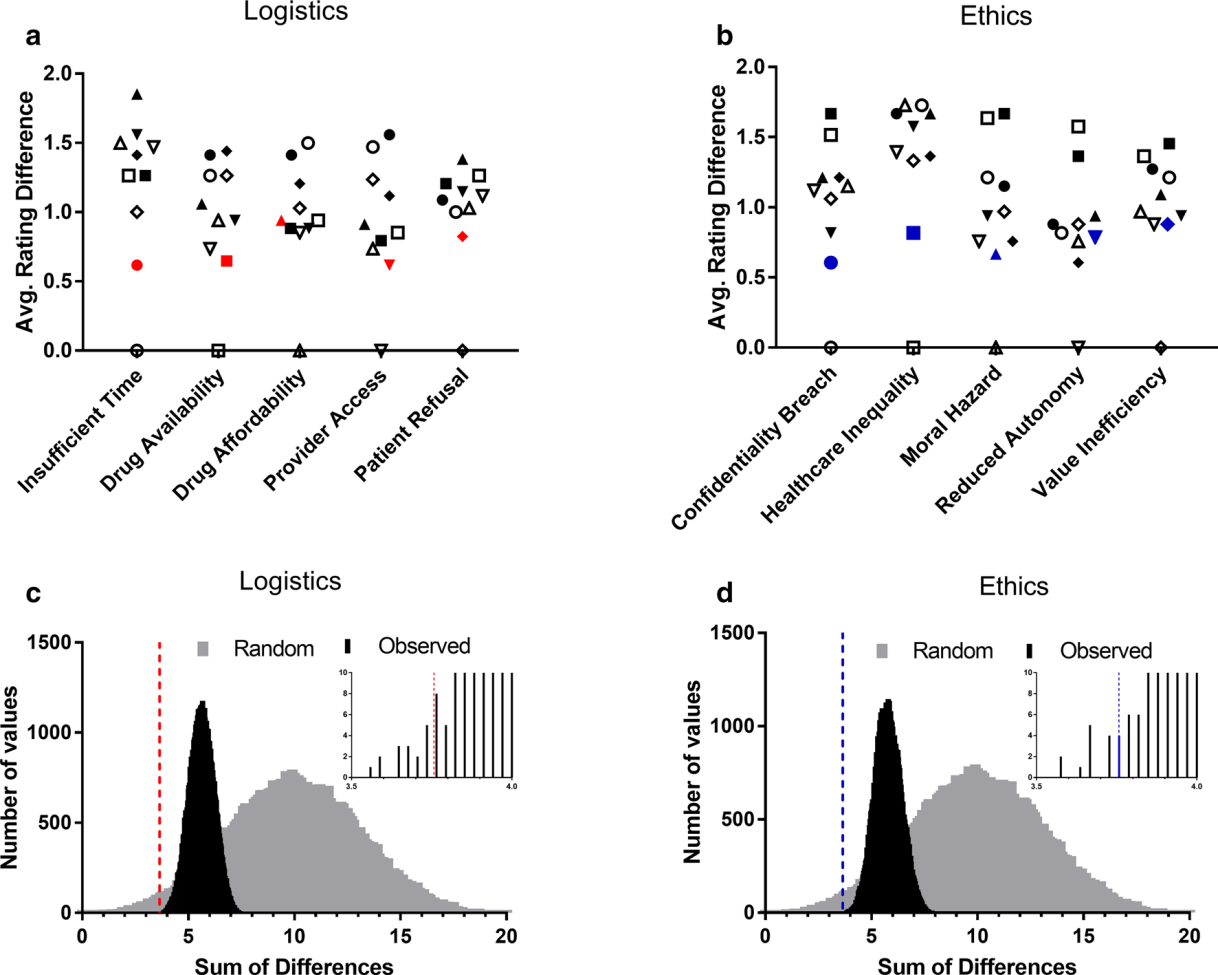
Average rating differences between pharmacist-reported logistical barriers (**a** ● = insufficient time, ■ = drug availability, ▲ = drug affordability, ▼ = provider access, ◆ = patient refusal) and ethical concerns (**b** ● = confidentiality breach, ■ = healthcare inequality, ▲ = moral hazard, ▼ = reduced autonomy, ◆ = value inefficiency). Filled shapes (●) correspond to current therapeutic responses while open shapes (○) correspond to anti-opioid vaccine responses. Colored shapes denote the five differences (●-○) in each matrix that were used to define the ‘reference equivalency’ model. Distribution of possible sums of differences in observed data and simulated random distribution for logistical barriers (**c**) and ethical concerns (**d**). Position of the sum of differences for the ‘reference equivalency’ model is denoted by the dashed lines, with subpanel zoomed in on the portion of the observed distribution where this value is found. Data plotted as average difference across all subjects (**a**, **b**) and counts of values falling within bins with a size of 0.1 (**c**, **d**)

Indeed, using the directly matched items presented to pharmacists, it was then considered whether pharmacists were considering anti-opioid vaccines as having equivalent logistical and ethical dimensions to current OUD therapeutics, a hypothetical cognitive approach referred to here as the ‘reference equivalency’ model. If application of this ‘reference equivalency’ cognitive model was the case, as hypothesized, the values provided by pharmacists should be most closely matched between identical items presented across both the current therapeutic and anti-opioid vaccine matrices, as compared to any other possible pairwise combination of these items, and not allowing each item to be matched with itself. That is, if the values for the five items in the logistical barrier matrix for current therapeutics are [●, ■, ▲, ▼, ♦], and the values given for the identical items in the logistical barrier matrix for anti-opioid vaccines are [○, □, △, ▽, ◇], then there are nine possible differences for each item in the matrix (e.g., ●-■, ●-▲, ●-▼, ●-♦, ●-○, ●-□, ●-△, ●-▽, ●-◇), resulting in 9^5^ possible sums of differences across all five items in the matrix. Using this notation, the ‘reference equivalency’ model is represented as [(●-○) + (■-□) + (▲-△) + (▼-▽) + (♦-◇)] (Fig. 7a, b).

To test whether this ‘reference equivalency’ model yields the smallest sum of differences for both logistical barriers and ethical concerns, the differences in values for each of the five items collected in the logistical barrier and ethical concern matrices, respectively, were summed to generate distributions of all 9^5^ possible resulting values (Fig. 7c, d). In this analysis, a smaller sum of differences represents a higher correlation between the paired combinations for current therapeutics and anti-opioid vaccines, demonstrating which items are most closely matched overall. With this analysis, the resulting means of the distributions for all sums of differences in each matrix (Logistics: 5.63 ± 0.60; Ethics: 5.78 ± 0.64) were significantly smaller than the sum of differences expected (10.00 ± 3.14; *p* < 0.0001 for both) if subjects were applying a random approach to consideration of these items overall.

Furthermore, when looking within the 9^5^ possible pairwise comparisons that were possible within the observed dataset, the sum of differences for the ‘reference equivalency’ cognitive model was among the smallest 0.0001% of all possible differences that could be generated for these two datasets, a result that is significantly different than expected by chance alone (Logistics: 3.65, Z = − 3.30, *p* < 0.001; Ethics: 3.65, Z = − 3.16, *p* = 0.0016). However, it should be noted that the ‘reference equivalency’ sum of differences for each domain was not the absolutely smallest of all possible sums of differences resulting from the observed data set. This finding indicates that while respondents were treating current OUD therapeutics and anti-opioid vaccines as having similar logistical and ethical dimensions overall, there were still specific areas that merited differential consideration between the two approaches. The two examined areas which differed most between current OUD therapeutics and anti-opioid vaccines were cost and patient refusal.

### Product-specific characteristics for anti-opioid vaccine development

When developing a new therapeutic approach, there are pragmatic considerations that arise regarding product development in addition to the broader logistical and ethical considerations considered above. To this end, students and pharmacists were presented with a matrix of questions related to vaccine product preferences across several dimensions. These dimensions included the generic considerations of cost and storage requirements, the vaccine-specific considerations of effective population coverage, time to onset of full protection, and duration of protection, and the product-specific consideration of breadth of opioid blockade (See Additional file 1: Figure S7). Students were asked fewer questions in this domain, given their relative lack of expected experience with product handling and recommendation. Furthermore, as a means to understand prioritization of broad-scale efforts, students and pharmacists were also asked to rank which of three domains were most important to consider in the development of VBTs. Across both populations, establishing efficacy was ranked significantly higher than management of either ethical or logistical considerations (*p* < 0.05 for all comparisons).

### Application of anti-opioid vaccines in ethically-variable clinical practice settings and populations

**Fig. 8:**
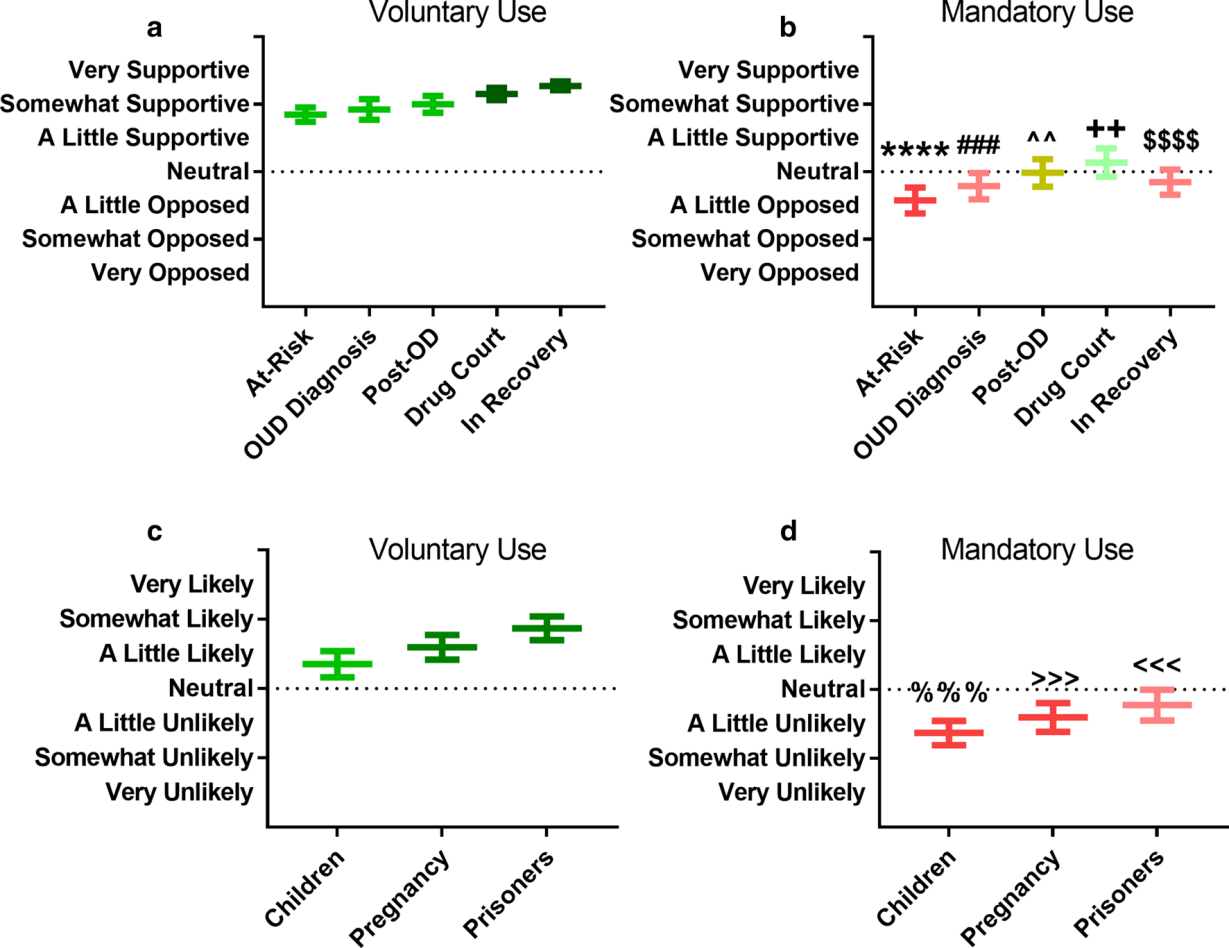
Pharmacist-reported support for the use of vaccine-based therapy across various clinical scenarios (**a**, **b**) and populations (**c**, **d**), under voluntary-use (**a**, **c**) and mandatory-use (**b**, **d**) scenarios. The dotted line on the figures indicates a true neutral response. All data plotted as mean ± SEM. ****p < 0.0001 vs. voluntary use in populations at-risk for OUD; ^###^*p* < 0.001 vs. voluntary use in populations with an OUD diagnosis with ongoing use;^^^^*p* < 0.01 vs. voluntary use in populations post-opioid overdose;^++^*p* < 0.01 vs. voluntary use in populations in drug court for opioid-related offences; ^$$$$^*p* < 0.0001 vs. voluntary in-recovery; ^%%%^*p* < 0.001 vs. voluntary use in children; ^>>>^*p* < 0.001 vs. voluntary use in pregnant individuals; ^<<<^*p* < 0.001 vs. voluntary use in prisoners

Determination of which clinical population to target with a given intervention is a critically important decision during the development of a new therapeutic intervention, as it informs the design of clinical trials, directly constrains the indications considered for approval by regulatory bodies, and often determines coverage access to the treatment itself. Selection of an appropriate population for anti-opioid vaccine use is potentially even more fraught than the average case, as considerations of mandated use and application to vulnerable populations have been a challenge in the context of both vaccination and substance use disorder treatment. One relevant example of mandatory therapy in a vulnerable population is court-ordered medical treatment subsequent to a drug possession and distribution offense; such policies lead to potentially coercive use of MAT for SUDs in incarcerated populations. These types of programs have been studied since the late 1990s and have grown substantially in the populations they serve [38, 39]. Therefore, we surveyed pharmacist-rated support or opposition for the mandatory and voluntary use of vaccine-based therapies across various clinical scenarios and in different vulnerable populations (Fig. 8). A global preference for voluntary use as compared to mandatory use was reported (*p* < 0.05 for all comparisons).

## Discussion

Broadly, these results suggest that while participant lack of familiarity with a given approach can be beneficial for acquiring opinions unbiased by prior exposure to that approach, this very lack of awareness may itself contribute to respondents’ self-exclusion from providing opinions on that topic. In this study, we attempted to address this concern through activation of expert identity and self-confidence by first presenting critical comparator approaches that the respondents indicated greater familiarity and direct experience with, prior to exposure to items regarding the less familiar approach [40–42]. To what degree this approach is required was not directly studied here, and further experiments looking to optimize respondent willingness to participate in early stakeholder engagement for drug development should consider the apparently opposing concerns of self-confidence activation and overall cognitive burden, as related to the use of comparator introductory items. In this dataset, expertise itself did not seem to be a strongly modifying factor for answers related to non-technical factors, such as overall OUD perceptions, outcome goals, and ethical considerations, as noted by the relatively high congruence between the student pharmacist and practicing pharmacist populations. Therefore, activation of self-confidence through general messaging that reinforces the opinion-based nature of the survey may be a useful approach.

Regarding these overall perceptions of OUD and the relative importance of individual patient outcomes, the fact that both pharmacists and pharmacy students rated overdose prevention as the most important of all the listed factors, and decreased reward as the least important is noteworthy for the viability of anti-opioid vaccine approaches. While such vaccines act both to decrease drug reward and overdose symptoms by limiting CNS exposure, it seems that the former function is of lower perceived utility (even if leading to abstinence) as compared to overdose prevention. To date, the clinical trials that have been undertaken with vaccination against cocaine and nicotine have focused on abstinence as their primary outcome, but these data indicate that anti-opioid vaccines may be better aligned with practitioner treatment outcome values if they instead focused on overdose prevention [43, 44]. When further considering the relative importance of societal outcomes, both pharmacists and pharmacy students again had similar preferences overall, with development of new treatments ranked as markedly less important than any of the other factors. It is possible that this represents an intrinsic bias toward ascribing preference to roles that are associated with self-identity; this interpretation is further supported by the fact that students also rated provider education as more important than practicing pharmacists did, reflecting their own status as practitioners-in-training. However, when considering the efficacy of specific interventions on promoting positive OUD health outcomes, pharmacy students did rank behavioral therapy as the most effective intervention (above more pharmacy-centric roles such as medication therapy and drug use monitoring), which indicates that this bias toward self-identity is not the only factor involved. Indeed, the relatively lower importance ascribed to new treatment development may simply reflect a recognition that the success of any novel treatment is reliant on patients gaining access to that treatment, given that treatment access was rated as the most significant challenge with current therapeutics overall. The relative importance of internally generated constructs (e.g. self-identity) versus external factors (e.g. structural barriers) when ascribing importance to specific interventions and outcomes will likely emerge as stakeholders with different functions and self-identities in regard to medication development are presented with these questions.

In addition to medication access being rated as the most significant logistical barrier for current therapeutics, access inequality was also rated as the most concerning ethical issue by pharmacists, further highlighting the overall importance of improving care delivery in OUD in pharmacy settings. Considering the similarity observed in the pattern of responses to logistical barriers and ethical concerns for current therapeutics and anti-opioid vaccines, these results suggest that new anti-opioid vaccine therapeutics in this space may be better received amongst the sample studied by employing an egalitarian access approach and minimizing logistical barriers to delivery. However, this will likely be easier said than done, as the perceived barriers to anti-opioid vaccine therapy are not all controlled by the same stakeholders. Indeed, the two logistical areas deemed to have the greatest potential difference for anti-opioid vaccines in comparison to current therapeutics were affordability and patient refusal, where the locus of control is with manufacturers/payers and patients, respectively. Considering that reduced autonomy was the ethical concern with the largest increase between anti-opioid vaccines and current therapeutics, the relatively high variability observed in regard to considerations of mandatory vaccination scenarios may reflect underlying tensions between a recognition of likely elevated rates of patient refusal and a competing recognition of patient’s rights to autonomous decision making.

Despite the wide variability in responses regarding their level of support for mandatory vaccination across multiple clinical scenarios, on average, pharmacists in this sample did still exhibit support for voluntary use overall and opposition to mandatory use in most scenarios. Within this broader finding, there is a noteworthy trend that is seen in both voluntary and mandatory vaccination scenarios—reported support for voluntary OUD vaccination appears to increase through a general course of OUD clinical progression. This generalized clinical course starts with a patient at risk for OUD, followed with an OUD diagnosis, eventually resulting in an opioid overdose, followed by presentation in drug court, and finally recovery, pharmacists were increasingly supportive of voluntary OUD vaccination at each of these stages. Using the same timeline progression, pharmacists’ initial opposition to mandatory vaccination decreased at each stage, finally crossing over into weak support for mandatory use in drug court. However, at the point of recovery, pharmacists’ favorability toward mandatory vaccination returned to opposition overall. Given this pattern of results, pharmacists’ default preference for supporting patients’ autonomous decision making seem to weaken as OUD risks progress throughout the course of the illness. However, once recovery is reached, autonomous decision making is again seen as preferable, and support for voluntary vaccination reaches its maximum, perhaps indicating an expectation for self-selection for treatment from those individuals who still perceive themselves to be at high risk for negative outcomes. Amongst vulnerable populations where opioid vaccination has been suggested for use, including children or adolescents at risk for OUD, pregnant women with OUD, and prisoners with OUD, the same clear preference for voluntary versus mandatory use can be seen as in when considering a general adult patient, with a notably large group of respondents opposed to use of opioid vaccination in children under either circumstance.

To our knowledge, this study represents the first attempt to get empirical knowledge about ethical concerns related to vaccine-based therapies for SUDs from relevant stakeholder populations. Given the noninfectious nature of SUDs, the ethical considerations for anti-drug abuse vaccines differ substantially than their counterparts directed against communicable diseases. The potential infringement on autonomy is often cited by opponents of vaccine-based SUD treatment technology [19]. With respect to this concern, pharmacists responded with a clear opposition of mandatory vaccination across clinical stages of OUD, indicating that patient awareness of such infringements may be variable over the course of a SUD. Another ethical dimension of vaccine development is concerned with cost vs. benefit. While pharmacists and students generally indicated that patient cost should be minimized and access should be maximized, further study regarding acceptance of specific health systems approaches to achieve these goals is required. A final concerning ethical dilemma with vaccine-based SUD treatments is the potential for vaccine administration leading to patients to use higher doses of abused drugs or switching from one abused drug to another. Pharmacists and students indicated a relatively low level of concern in this ethical dimension. Future studies should aim to assess other relevant stakeholders whose perspectives and experiences may allow them to better speak to the degree which vaccine-based technology may result in the promotion of risky behaviors.

In addition to its clear role in ethical considerations for anti-opioid vaccine implementation, the question of mandatory use may also have follow-on logistical impacts influencing treatment availability. Amongst all interventions, including both pharmacologic and non-pharmacologic approaches, the PDMP is the only one that is legally mandated to be used in the state of Wisconsin [44]. Perhaps, unsurprisingly then, the PDMP was found to be the most available and most used resource by both pharmacy students and practicing pharmacists in Wisconsin, with 100% availability reported by active practitioners. While similar drug use monitoring software systems exist in virtually all other states, their legally mandated use varies quite dramatically [45]. The difference in legal status may have important implications for both availability and use when applying this survey approach to populations in other states. Current medication options for OUD were reported to be available in 60–80% of student pharmacist workplaces, with buprenorphine/naloxone combinations being the most frequently used medication. Pharmacists reported similar rates of availability for non-injectable formulations of these medications, although their reported use frequency was lower in general. This difference in reported frequency between the two samples is potentially due to different roles within the pharmacy based on career stage between students and pharmacists. Aside from the PDMP, the availability of other non-medication interventions was reported as being similar to the availability of non-injectable OUD medications. Another notable finding in regard to pragmatic implementation of anti-opioid vaccines is that injectable formulations of OUD medications were all less available than their non-injectable counterparts. Injectable medications were also less frequently used, although this is directly impacted by differences in dosing frequency between the formulations.

Beyond highlighting this potential value to be gained by considering non-injectable vaccine formulations, several other responses in regard to vaccine product characteristics were also identified in this study. Students and practicing pharmacists appeared to agree that an anti-opioid vaccine would ideally have an onset of action within weeks at most. Likewise, pharmacists indicated a desired duration of coverage would be a period of more than a year. Both groups also endorsed desiring vaccines with broad coverage against multiple opioids. This approach has been attempted preclinically, but the breadth of coverage desired by pharmacists is even greater than the widest breadth of coverage that has been attempted to date [47–50]. Pharmacists in this sample demonstrated less concern with supply chain and handling logistics for anti-opioid vaccines, in comparison to other factors, likely reflecting the resource-rich environment in which they practice; this consideration would likely differ in a broader, more global sample. Finally, in regard to product performance, there was a reasonable degree of tolerance for treatment failure in a subset of vaccinated individuals, although it is presently unclear whether the source of high variability in this measure is a result of intrinsic differences in risk tolerance, or a reflection of further need to refine the language used for this relatively complicated concept. Considering that previous trials of small molecule vaccination against nicotine and cocaine have exhibited substantial inter-individual variability in antibody titer production, this remains a major area of need in regard to achieving approval of anti-opioid vaccines as an efficacious approach [43, 44].

As might be expected from individuals trained to practice evidence-based medicine, pharmacy students and pharmacists both agreed that their primary concern with anti-opioid vaccine development was demonstrating efficacy, rather than ethical or logistical concerns. It remains to be seen whether this opinion is also held by other stakeholder groups, but this endorsement of an efficacy-first approach certainly speaks to the continued central role that pre-clinical and clinical researchers will have in terms of drug development, even if broader stakeholder engagement approaches become more widespread. And overall, despite their reported unfamiliarity with anti-opioid vaccines, both students and pharmacists seemed to have a relatively high degree of optimism about the possible outcomes of this process, as they were overwhelmingly positive in regard to the potential of anti-opioid vaccines to become helpful interventions for OUD. However, while the iterative nature of this study was geared toward helping promote neutrality of the final survey (including exploration of both unipolar and bipolar scale approaches for response anchoring, altered balance of number of items in each survey-sub-section, use of thematically-related distractor domains such as OUD resource use, and the inclusion of potentially ethically challenging scenarios immediately prior to this summary to provide a countervailing influence in the form of recency bias) [51], this positive bias may still be influenced by unintended survey demand characteristics [52–54].

A final discussion of strengths and limitations of this study more broadly is also warranted, as this report is not only the first survey of practitioner opinions regarding anti-opioid vaccines as a potential future intervention, it is apparently among the first to even indirectly address whether pharmacy practitioner experiences, priorities, and opinions can be reliably assessed in relation to early stage drug development research. In regard to this broader potential relevance, we believe that the use of anti-opioid vaccines as the test case for this initial study provides several important strengths when considering generalizability of this overall approach. Firstly, the nature of the anti-opioid vaccine intervention allowed for assessment across both logistical and ethical domains, which are likely to independently inform successful implementation of novel technologies. Secondly, there has been recent expansion in pharmacist professional activities surrounding opioid medications, including the widespread adoption of pharmacist naloxone dispensing per protocol, and expanding authority to administer long-acting injectable medications like naltrexone. These recent changes provide a useful comparator group in regard to opinions regarding disruption of usual workflow and the attendant results arising from them, in order to distinguish opinion domains unique to a given intervention versus those reflective of more general concerns overall. Finally, the large burden of OUD and opioid overdose cases in the US at the time of this study promoted large-scale efforts to promote awareness of the issue among the target population, thus potentially providing a more homogenous background level of consideration of the topic than would otherwise be the case. In regard to limitations, in addition to the risk of establishing subconscious demand characteristics as noted above, the small sample size and confined geographic location of the study may introduce additional response biases and limit the overall generalizability of the findings. Additionally, as survey refinement was an intrinsic goal of the study process, differently worded or scaled questions were asked of students and pharmacists, so assessment of student populations with the final version of the survey would be beneficial for making direct comparisons. Finally, and most importantly, the consideration of only pharmacists and pharmacy students in this study necessarily eliminates important, and likely differing, opinions from other classes of practitioner and non-practitioner stakeholders. For this reason, administration of this instrument to other stakeholder groups is a critical priority for validation of the findings described here.

## Conclusions

Pre-clinical research has demonstrated that bioconjugate vaccines against small molecules can consistently block both the therapeutic and euphoric effects of drugs of abuse in rodent models, but many unresolved questions remain in regard to logistical implementation and ethical use of anti-opioid vaccines in human population. In advance of clinical trials studying vaccine-based therapies for OUD, engagement of pharmacy stakeholders was able to successfully characterize practitioner opinions in regard to ethical, logistical, and clinical considerations. Despite differing levels of direct clinical experience, pharmacy students and practicing pharmacists exhibited broadly similar opinions concerning current availability and utilization of resources used to manage OUD, desirable characteristics of anti-opioid vaccine produces, and perceived barriers to implementation. Subject responses in regard to hypothetical anti-opioid vaccine treatments they were unfamiliar with tended to adhere to their responses in regard to known treatment options within the same domain, while nevertheless taking account of salient differences between the treatment modalities, such as potential cost, patient preferences, and concerns about autonomous decision making. Assessment of product-specific domains identified time to onset, duration of treatment, and breadth of opioid coverage as areas with more demanding expectations in comparison to the current state of the art. Analysis of support for use in adult populations indicates that voluntary vaccination of individuals currently in recovery from OUD is likely to be the most well-supported population for clinical intervention. Mandatory vaccination was opposed on average, aside from in the context of drug court. Vaccination within protected populations, such as children, pregnant women, and prisoners was only supported when voluntary. Beyond these differences among protected populations, additional subgroups are worthy of study in future survey efforts. For example, there may be meaningfully different levels of vaccine acceptance across relevant subpopulations such as veteran, Medicare, or Medicaid patients. The studied subjects indicated that anti-opioid vaccines would be potentially helpful overall, but the degree to which pharmacist and pharmacy student opinions on anti-opioid vaccines align with those of other healthcare practitioners, payers, patients, regulators, and researchers remains an important question for future study.

## Supplementary Information

**Additional file 1.** Description of Data: Supplemental Methods, Figures S1–S7, and Appendices A–C.

## Data Availability

Study data were collected on the REDCap database hosted by the University of Wisconsin – Madison School of Medicine (https://redcap.ictr.wisc.edu/). Public access to this database is closed. The authors received administrative permission to access and use the site for initial dataset capture. All datasets collected during the current study are now available in the Mendeley Data repository (https://data.mendeley.com/). Public access to this database is open. The published dataset is entitled “Wenthur, Cody; Stewart, Amy; Wartenweiler, Vincent; Chung, Grace (2021), “Data for: Pharmacy Stakeholder Reports on Ethical and Logistical Considerations in Anti-Opioid Vaccine Development”, Mendeley Data, V1” (https://doi.org/10.17632/2zv856bysz.1). All datasets used and analyzed during this study are also available from the corresponding author on reasonable request.

## Abbreviations

OUD: Opioid use disorder
SUD: Substance use disorders
SAMHSA: Substance Abuse and Mental Health Services Administration
MAT: Medication-assisted treatment
PDMP: Prescription drug monitoring program
IRB: Institutional Review Board
CTSA: Clinical and Translational Science Award
NIH: National Institutes of Health
NCATS: National Center for Advancing Translational Sciences
