# Supplementary material for: Pharmacy stakeholder reports on ethical and logistical considerations in anti-opioid vaccine development
Source: BMC Med Ethics. 2021 Mar 25;22:30. doi: 10.1186/s12910-021-00599-2 (PMC7992836; doi:10.1186/s12910-021-00599-2)
Supplement: Supplementary file 1 — Additional file 1. Description of Data: Supplemental Methods, Figures S1–S7, and Appendices A–C. [file 12910_2021_599_MOESM1_ESM.pdf]

Pharmacy Stakeholder Reports of Ethical and Logistical Considerations in Anti-Opioid Vaccine Development

SUPPLEMENTAL INFORMATION

Table of Contents

A. Supplemental Methods
    i. Student survey details
    ii. Pharmacist survey details
B. Supplemental Figures
    i. Word similarity analysis
    ii. Cross-tabulation frequency table analysis **1**
    iii. Cross-tabulation frequency table analysis **2**
    iv. Cross-tabulation frequency table analysis **3**
    v. Student perceptions on OUD severity and treatments
    vi. Student perceptions of ethical and logistical concerns
    vii. Desired vaccine characteristics and prioritization of development domains
C. Appendix A. Final Student Pharmacist REDCap Survey.
D. Appendix B. Final Practicing Pharmacist REDCap Survey.
E. Appendix C. Focus group guide sheet.

A. SUPPLEMENTAL METHODS

i. Student survey

Using feedback from the pilot survey, a final student survey was developed that contained selected Likert scale, rank order, and radio button questions regarding the same topics as covered in the pilot survey. The final student survey contained 21 items and required roughly 60 physical “clicks” (Supplemental Information). Inclusion and exculsion criteria were the same as the focus group, aside from the exclusion of DPH-4 students and those who participated in the focus group and/or pilot survey. The student survey was administered through REDCap. Each class of current students received an in-person announcement from a study team member before or after a well-attended class including an email link to participate. A chance to win a gift card was provided as incentive for those individuals who chose to provide an email address in a separate database that opened upon completion of the survey. The survey was open to participants for three weeks with reminder emails sent weekly until the surveys were closed.

ii. Practitioner survey

Using feedback from current practitioners in regard to the contents of the student survey, the pharmacist survey was generated that contained Likert scale, rank order, and radio button questions regarding the same content areas as the student survey. The final pharmacist survey contained 29 items and required roughly 89 physical “clicks” (Supplemental Information). Currently licensed pharmacists were eligible to participate in the survey. Pharmacists were contacted via the Pharmacy Practice Enhancement and Action Research Link (PearlRx), a practice-based research network of pharmacists . The pharmacist survey was administered through REDCap. A chance to win a gift card was provided as incentive for those individuals who chose to provide an email address in a separate database that opened upon completion of the survey. The survey was open to participants for three weeks with reminder emails sent weekly until the surveys were closed

B. SUPPLEMENTAL FIGURES

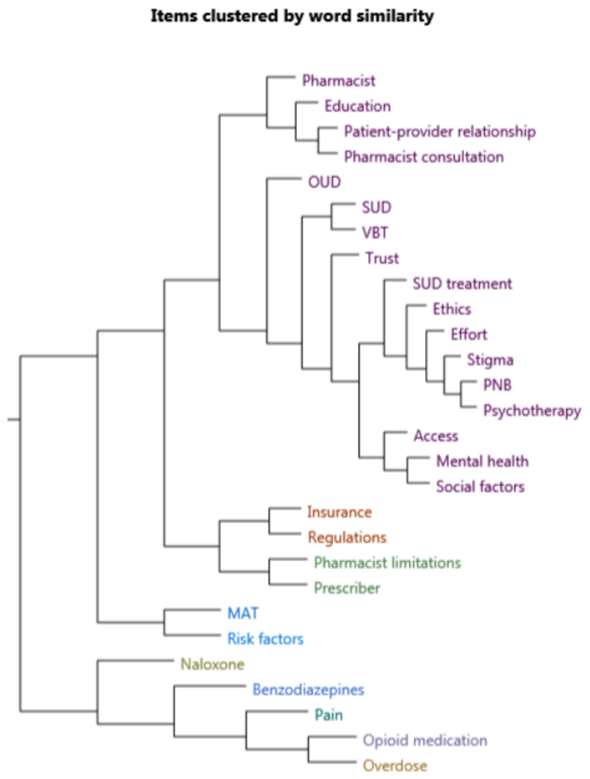

Supplemental Figure 1. Word similarity analysis completed using the word similarity query in QSR NVivo. Visually represents how closely participants interrelated different themes of discussion.

	Patient-provider relationship	Pharmacist	Pharmacist consultation	Pharmacist limitations	Prescriber	Trust	**Total**
Participant 1	0%	5.26%	0%	42.86%	12.50%	11.11%	10.48%
Participant 2	25%	26.32%	7.14%	14.29%	20.83%	22.22%	12.10%
Participant 3	25%	42.11%	35.71%	14.29%	41.67%	44.44%	41.13%
Participant 4	50%	15.79%	14.29%	14.29%	8.33%	11.11%	12.10%
Participant 5	0%	10.53%	42.86%	14.29%	16.67%	11.11%	24.19%
**Total**	100%	100%	100%	100%	100%	100%	100%

Supplemental Figure 2. Cross-tabulation frequency table analysis completed using the crosstab query in QSR NVivo. Displays the frequency that specific participants spoke about given patient and prescriber factors and how frequently specific participants spoke overall.

	Trust	Mental health	Psychotherapy	SUD treatment	**Total**
Participant 1	11.11%	4.35%	8.70%	9.09%	7.95%
Participant 2	22.22%	17.39%	21.74%	12.12%	17.05%
Participant 3	44.44%	34.78%	39.13%	39.39%	38.64%
Participant 4	11.11%	21.74%	21.74%	9.09%	15.91%
Participant 5	11.11%	21.74%	8.70%	30.30%	20.45%
**Total**	100%	100%	100%	100%	100%

Supplemental Figure 3. Cross-tabulation frequency table analysis completed using the crosstab query in QSR NVivo. Displays the frequency that specific participants spoke about given factors concerning trust in the mental healthcare industry and how frequently specific participants spoke on the topics overall.

	Naloxone	Overdose	Risk factors	Total
Participant 1	33.33%	0%	0%	9.09%
Participant 2	0%	0%	0%	0%
Participant 3	66.67%	100%	66.67%	81.82%
Participant 4	0%	0%	0%	0%
Participant 5	0%	0%	33.33%	9.09%
**Total**	100%	100%	100%	100%

Supplemental Figure 4. Cross-tabulation frequency table analysis completed using the crosstab query in QSR NVivo. Displays the frequency that specific participants spoke about various factors influencing overdose and its management and how frequently specific participants spoke on the topics overall.

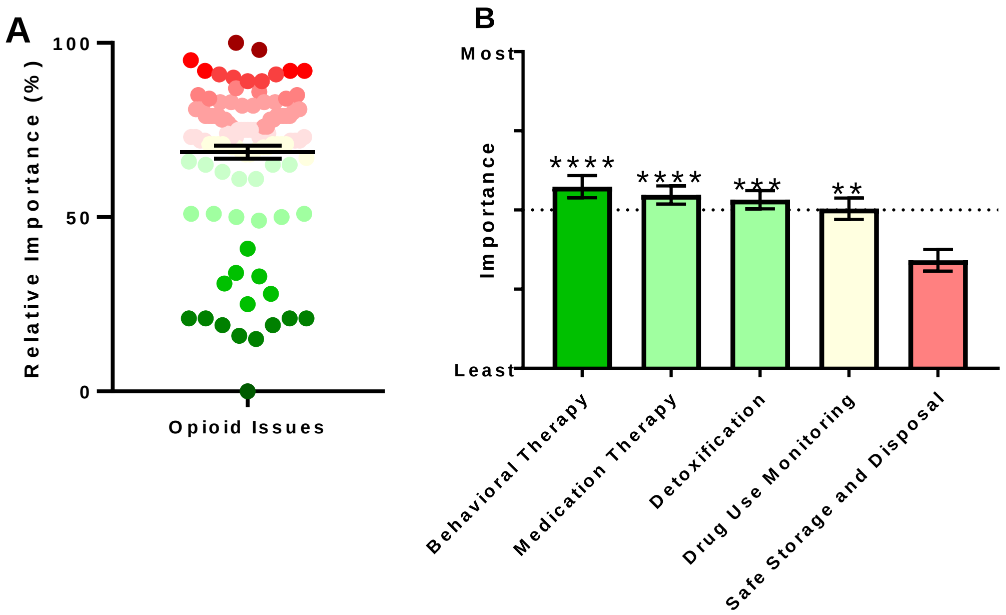

Supplemental Figure 5. Student-reported perceptions of national importance of OUD as compared to other healthcare issues (A). Student-reported ranking of current topics for managing OUD (B). The dotted line indicates the scale midpoint. All data plotted as mean ±SEM. ANOVA with Tukey: ** $p<0.01$ vs. Safe Storage; *** $p<0.001$ vs. Safe Storage; **** $p<0.0001$ vs. Safe Storage.

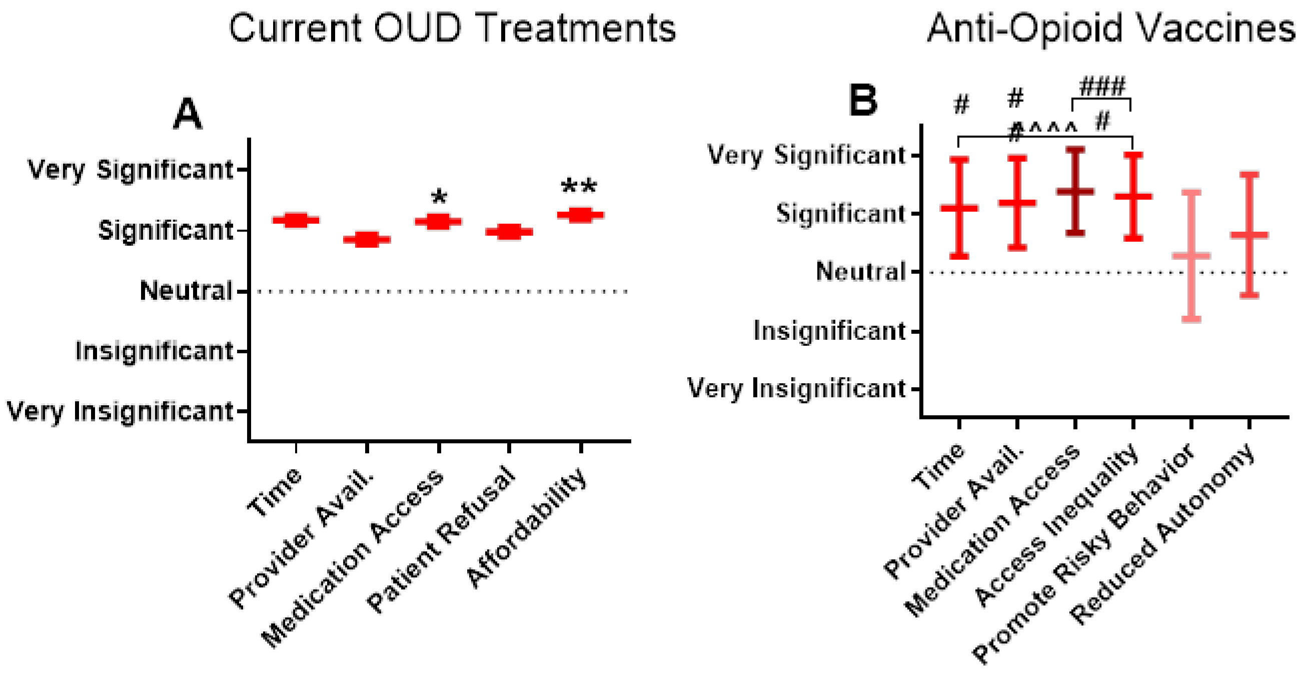

Supplemental Figure 6. Student-reported perceptions regarding logistical barriers and ethical concerns for current treatments (A) and anti-opioid vaccines (B). Dotted line indicates neutral response. All data plotted as mean ± SEM. Friedman with Dunn's: * p<0.05 vs. provider availability; ** p<0.01 vs. provider availability; # p<0.05 vs. reduced autonomy; ## p<0.01 vs. reduced autonomy; #### p<0.0001 vs. reduced autonomy; ^^^^ p<0.0001 vs. promotion of risky behavior.

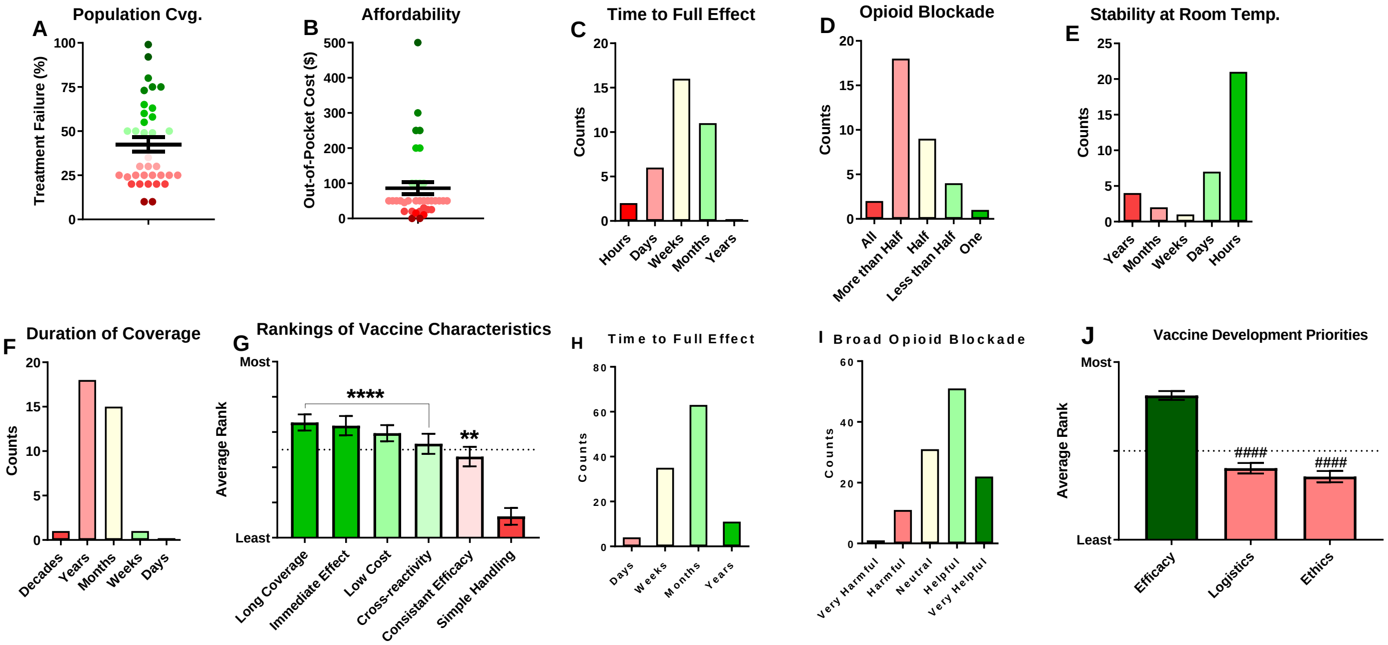

Supplemental Figure 7. Pharmacist-reported acceptable characteristics (A-F) and overall ranked importance of general factors for anti-opioid vaccines (G). Student-reported acceptable characteristics of anti-opioid vaccines (H and I). Aggregate pharmacist and student-reported rankings of priorities concerning anti-opioid vaccine development (J). Data in A, B, G, J plotted as mean ±SEM. ANOVA with Tukey's: ** $p<0.05$ vs. simple handling; **** $p<0.0001$ vs. simple handling; #### $p<0.0001$ vs. efficacy.

B. APPENDIX A

Pharmacy Student Survey

Please complete the survey below.

Thank you!

Demographic information

Are you at least 18 years of age or older?
- ◯ Yes
- ◯ No

What year are you in pharmacy school?
- ◯ DPH-1 ◯ DPH-2 ◯ DPH-3
- ◯ DPH-4

Practice information

How much time have you spent working in a healthcare setting?
- ◯ None
- ◯ Less than 1 year
- ◯ Between 1-3 years
- ◯ Between 3-5 years
- ◯ Between 5-10 years
- ◯ Between 10-20 years
- ◯ 20 years or more

In which healthcare setting do you CURRENTLY work?
- ◯ Outpatient (e.g. Community, Clinic, Ambulatory Care)
- ◯ Inpatient (e.g. Hospital, Institutional, Long-term Care)
- ◯ Other (e.g. Non-patient Care, Academia, Pharmaceutical Industry)

Opinions on Opioid Use Disorder (OUD)

How would you rate the IMPORTANCE / HEALTH IMPACT of improved Opioid Use Disorder (OUD) treatment NATIONALLY, as compared to other healthcare issues ? (i.e. If you marked 5%, it would mean that you would think improving OUD is in the top 5% of the national healthcare issue).

0 — 50 — 100

(Place a mark on the scale above)

RANK the individual changes below from MOST to LEAST IMPORTANT for optimizing OUD outcomes.

	1 (Most Important)	2	3	4	5 (Least Important)
Increased drug abstinence	◯	◯	◯	◯	◯
Increased social function/integration	◯	◯	◯	◯	◯

Decreased drug craving	○	○	○	○	○
Decreased drug overdose	○	○	○	○	○
Decreased drug reward	○	○	○	○	○

RANK the societal / structural changes below from MOST to LEAST IMPORTANT for optimizing OUD outcomes.

	1 (most important)	2	3	4 (least important)
Access to/use of current OUD treatments	○	○	○	○
Development of new treatments for OUD	○	○	○	○
Opioid prescribing patterns	○	○	○	○
Public/patient education on OUD	○	○	○	○

Current OUD Intervention Utilization

How familiar are you with current interventions for OUD?

- ○ Very familiar
- ○ Familiar
- ○ Neutral
- ○ Unfamiliar
- ○ Very unfamiliar

About how frequently do you use each of the CURRENT INTERVENTIONS below for management of OUD at your primary practice site?

	Not available	Hourly (or More)	Daily	Weekly	Monthly (or Less)	Never
Behavioral therapy	○	○	○	○	○	○
Buprenorphine/Naloxone (Suboxone)	○	○	○	○	○	○
Controlled Substance Monitoring Database	○	○	○	○	○	○
Detoxification support	○	○	○	○	○	○
IM long-acting Naltrexone (Vivitrol)	○	○	○	○	○	○
Medication-assisted therapy (MAT)	○	○	○	○	○	○
Medication drop box	○	○	○	○	○	○
Medication therapy management (MTM)	○	○	○	○	○	○
Naloxone	○	○	○	○	○	○
Oral Naltrexone (Revia)	○	○	○	○	○	○
Urine drug screen	○	○	○	○	○	○

RANK the CURRENT TOPICS below from MOST to LEAST IMPORTANT in management of OUD.					
	1 (most important)	2	3	4	5 (least important)
Behavioral therapy	○	○	○	○	○
Controlled substance use monitoring	○	○	○	○	○
Detoxification support	○	○	○	○	○
Medication-assisted therapy (MAT)	○	○	○	○	○
Safe medication storage and disposal	○	○	○	○	○

How helpful / unhelpful are CURRENT INTERVENTIONS in terms of SUPPORTING OUTCOMES below?					
	Very Helpful	Helpful	Neutral	Unhelpful	Very Unhelpful
Increased drug abstinence	○	○	○	○	○
Increased social function/integration	○	○	○	○	○
Decreased drug craving	○	○	○	○	○
Decreased drug overdose	○	○	○	○	○
Decreased drug reward	○	○	○	○	○

How significant / insignificant are the LOGISTICAL BARRIERS below when implementing CURRENT INTERVENTIONS for OUD?					
	Very Significant	Significant	Neutral	Insignificant	Very Insignificant
Insufficient time/facilities	○	○	○	○	○
Patient refusal/resistance	○	○	○	○	○
Provider availability/education	○	○	○	○	○
Treatment affordability/reimbursement	○	○	○	○	○
Treatment availability/access	○	○	○	○	○

In comparison to interventions for other therapeutic areas, how well / poorly are CURRENT INTERVENTIONS FOR OUD meeting the needs of ALL STAKEHOLDERS AT YOUR PRIMARY PRACTICE SITE?

- ○ Much Better
- ○ Better
- ○ Similarly
- ○ Worse
- ○ Much Worse

Vaccine Based Therapeutics for OUD

How familiar are you with the concept of vaccine-based therapies (VBTs)?

- ○ Very familiar
- ○ Familiar
- ○ Neutral
- ○ Unfamiliar
- ○ Very unfamiliar

VBTs for OUD

Recently, there have been new advances in vaccines used to treat opioid use disorder. For example, one vaccine in the pre-clinical stage aims to program the body to create antibodies to heroin. The concept supporting these vaccines is that when someone who has been vaccinated uses an opioid, the antibodies will selectively bind to that target and prevent the drug from entering the CNS and binding to central opioid receptors.

The following questions are designed to gather your opinions regarding the preferred characteristics and applications of a potential OUD vaccine product.

VBT for OUD may not induce lifetime coverage, unlike other traditional vaccines. Continued protection could require additional booster injections.

What is the minimum coverage time from a single injection that would be ACCEPTABLE for use in your PRIMARY PRACTICE SETTING?

- ○ Days (or Shorter)
- ○ Weeks
- ○ Months
- ○ Years (or Longer)

VBT for OUD may be designed to cover a broad range of opioids, PREVENTING the abuse (or therapeutic use) of any opioid.

How helpful / harmful would such BROAD coverage be for treatment of patients in your PRIMARY PRACTICE SETTING?

- ○ Very Helpful
- ○ Helpful
- ○ Neutral
- ○ Harmful
- ○ Very Harmful

How supportive / opposed would you be of VBT use in PATIENTS under the circumstances below?

	Very Supportive	Supportive	Neutral	Opposed	Very Opposed
Drug court sentencing	○	○	○	○	○
Hospital discharge following overdose	○	○	○	○	○
In recovery from OUD and currently abstaining	○	○	○	○	○
Prophylactic use with elevated OUD risk/family history	○	○	○	○	○
Recent diagnosis of OUD with ongoing use	○	○	○	○	○
Universal prophylactic use	○	○	○	○	○

How significant/insignificant do you think the following BARRIERS would be when implementing VBT for OUD?

	Very Significant	Significant	Neutral	Insignificant	Very Insignificant
Insufficient time/facilities	○	○	○	○	○
Provider availability/education	○	○	○	○	○
Treatment availability/access	○	○	○	○	○

**How concerned/unconcerned are you regarding the following ETHICAL ISSUES for VBT in OUD?**					
	Highly concerned	Concerned	Neutral	Unconcerned	Highly unconcerned
Costs/access inequality	○	○	○	○	○
Moral hazard/promoting risky behavior	○	○	○	○	○
Patient autonomy	○	○	○	○	○

**RANK the following areas of consideration in regard to future implementation of VBT for OUD, from MOST to LEAST IMPORTANT for further study.**			
	1 (Most Important)	2	3 (Least Important)
Efficacy	○	○	○
Ethics	○	○	○
Logistics/integration of care	○	○	○

In your opinion, how helpful/unhelpful would VBT be as an addition to currently available OUD interventions, OVERALL?

- ○ Very Helpful
- ○ Helpful
- ○ Neutral
- ○ Unhelpful
- ○ Very Unhelpful

Thank you very much for taking the time to complete this survey!

C. APPENDIX B

Pharmacy Survey

Please complete the survey below.

Thank you!

**Practice information**	
Are you an ACTIVELY-LICENSED pharmacist?	◯ Yes<br>◯ No
In which setting is your CURRENT PRIMARY PRACTICE site?	◯ Outpatient (e.g. Community, Clinic, Ambulatory Care)<br>◯ Inpatient (e.g. Hospital, Institutional, Long-term Care)<br>◯ Other (e.g. Non-patient Care, Academia, Pharmaceutical Industry)<br>(If you are currently retired or between positions, select the option that reflects your most recent employment)
How many YEARS have you worked at your CURRENT PRIMARY PRACTICE site?	◯ 0-4 years<br>◯ 5-9 years<br>◯ 10-14 years<br>◯ 15-20 years<br>◯ More than 20 years
What PERCENTAGE OF YOUR WORKDAY is generally spent addressing the THERAPEUTIC USE OR MISUSE OF OPIOIDS in your patients?	0 50 100<br>*(Place a mark on the scale above)*
CHECK ANY of the following choices that describe your MAJOR RESPONSIBILITIES at your current practice site.	☐ Pain Management<br>☐ Substance Use Disorder / Addiction Therapy<br>☐ Palliative Care

Opinions on Opioid Use Disorder (OUD)

RANK the PATIENT OUTCOMES below from MOST to LEAST IMPORTANT for management of OUD and its associated risks.

	1 (Most Important)	2	3	4	5 (Least Important)
Increased drug abstinence	◯	◯	◯	◯	◯
Increased social function	◯	◯	◯	◯	◯
Decreased drug craving (a.k.a. need)	◯	◯	◯	◯	◯
Decreased drug overdose	◯	◯	◯	◯	◯

Decreased drug reward (a.k.a. euphoria)	○	○	○	○	○

RANK the SOCIETAL OUTCOMES below from MOST to LEAST IMPORTANT for management of OUD and its associated risks.

	1 (most important)	2	3	4 (least important)
Increased access to current OUD treatments	○	○	○	○
Increased development of new treatments for OUD	○	○	○	○
Increased provider education on appropriate opioid prescribing	○	○	○	○
Increased public education on risk factors for OUD	○	○	○	○

Current OUD Intervention and Resource Utilization

How FAMILIAR are you with current RESOURCES AND MEDICATIONS for managing OUD and its associated risks?

○ Extremely familiar
○ Very Familiar
○ Somewhat Familiar
○ Slightly Familiar
○ Not at All Familiar

How FREQUENTLY do you use each of the RESOURCES below for managing OUD and its associated risks in your patients?

	Not Available to Me	Available, but Never Use	Monthly or Less	Weekly	Daily or More
Behavioral Therapies (e.g. CBT, Contingency Mgmt)	○	○	○	○	○
Controlled Substance Monitoring Database (e.g. PDMP)	○	○	○	○	○
Medication Dropbox	○	○	○	○	○
Medication Take-Back Program	○	○	○	○	○
Technology Assisted Interventions (e.g. Mobile health apps)	○	○	○	○	○
Urine Drug Screening	○	○	○	○	○

How FREQUENTLY do you use each of the MEDICATIONS below for managing OUD and its associated risks in your patients?

	Not Available to Me	Available, but Never Use	Monthly or Less	Weekly	Daily or More
Injectable Buprenorphine (e.g. Sublockade)	○	○	○	○	○
Buccal/Sublingual Buprenorphine (e.g. Subutex, Suboxone)	○	○	○	○	○
Injectable Naltrexone (e.g. Vivitrol)	○	○	○	○	○
Oral Naltrexone (e.g. Revia)	○	○	○	○	○
Injectable Methadone	○	○	○	○	○
Oral Methadone	○	○	○	○	○
Injectable Naloxone (e.g. Evzio)	○	○	○	○	○
Intranasal Naloxone (e.g. Narcan)	○	○	○	○	○

How SIGNIFICANT are the BARRIERS below when using currently available MEDICATIONS for management of OUD and its associated risks?

	Not at All Significant	Slightly Significant	Somewhat Significant	Very Significant	Extremely Significant
Insufficient time	○	○	○	○	○
Lack of provider availability	○	○	○	○	○
Lack of treatment affordability	○	○	○	○	○
Lack of treatment access	○	○	○	○	○
Patient refusal	○	○	○	○	○

How CONCERNING are the ETHICAL ISSUES below when using currently available MEDICATIONS for management of OUD and its associated risks?

	Not at All Concerning	Slightly Concerning	Somewhat Concerning	Very Concerning	Extremely Concerning
Confidentiality breaches	○	○	○	○	○
Access inequality	○	○	○	○	○
Promotion of risky behavior	○	○	○	○	○
Reduced patient autonomy	○	○	○	○	○
Inefficient use of scarce resources	○	○	○	○	○

IN COMPARISON TO MEDICATIONS AVAILABLE FOR OTHER THERAPEUTIC AREAS, how are currently available medications for management of OUD and its associated risks meeting your practice needs?

- ○ Much Better
- ○ Somewhat Better
- ○ A Little Better
- ○ Similarly
- ○ A Little Worse
- ○ Somewhat Worse
- ○ Much Worse

**Vaccine Based Therapeutics (VBTs) for OUD**	
How FAMILIAR are you with the concept of vaccine-based therapeutics (VBTs) for management of OUD and its associated risks?	◯ Extremely familiar<br>◯ Very Familiar<br>◯ Somewhat Familiar<br>◯ A Little Familiar<br>◯ Not at All Familiar

VBTs for OUD
Recently, there have been new advances in vaccines used to treat opioid use disorder. For example, one vaccine in the pre-clinical stage aims to program the body to create antibodies to heroin. The concept supporting these vaccines is that when someone who has been vaccinated uses an opioid, the antibodies will selectively bind to that target and prevent the drug from entering the CNS and binding to central opioid receptors.

The following questions are designed to gather your opinions regarding the preferred characteristics and applications of a potential OUD vaccine product.

VBT for OUD may require patients to get multiple injections before full efficacy is achieved, akin to traditional vaccines.<br><br>What is the MAXIMUM TIME TO ACHIEVE FULL IMMUNOLOGIC COVERAGE THAT WOULD BE ACCEPTABLE for use at your primary practice site?	◯ Hours<br>◯ Days<br>◯ Weeks<br>◯ Months<br>◯ Years
VBT for OUD may achieve full efficacy in only a subset of the vaccinated population, akin to traditional vaccines.<br><br>What is the MINIMUM PERCENTAGE OF PATIENTS RECEIVING PROTECTION from OUD and its associated risks THAT WOULD BE ACCEPTABLE for use at your primary practice site?	0 50 100<br>*(Place a mark on the scale above)*
VBT for OUD may incur out of pocket costs to patients, akin to other prescription medications.<br><br>What is the AVERAGE OUT-OF-POCKET COST to a patient (in US $) THAT WOULD BE ACCEPTABLE for a SINGLE VBT INJECTION at your primary practice site?	________________________
VBT for OUD may require special handling, including maintenance of a documented cold chain, akin to traditional vaccines.<br><br>What is the MINIMUM DURATION OF STABILITY AT ROOM TEMPERATURE THAT WOULD BE ACCEPTABLE for use at your primary practice site?	◯ Hours<br>◯ Days<br>◯ Weeks<br>◯ Months<br>◯ Years
VBT for OUD may not induce lifetime coverage, unlike other traditional vaccines. Continued protection could require additional booster injections.<br><br>What is the MINIMUM FREQUENCY BETWEEN BOOSTER INJECTIONS THAT WOULD BE ACCEPTABLE for use at your primary practice site?	◯ Days<br>◯ Weeks<br>◯ Months<br>◯ Years<br>◯ Decades

VBT for OUD may be designed to block the actions of a broad range of opioids, which would PREVENT BOTH THERAPEUTIC USE AND MISUSE of each blocked opioid.

What APPROXIMATE BREADTH OF OPIOID BLOCKADE would be MOST BENEFICIAL for treatment of patients at your primary practice site?

- ○ Blockade of All Known Opioids
- ○ Blockade of More than Half of Known Opioids
- ○ Blockade of Half of Known Opioids
- ○ Blockade of Less than Half of Known Opioids
- ○ Blockade of a Single Known Opioid

RANK each of the following VBT PRODUCT CHARACTERISTICS for management of OUD and its associated risks, from MOST to LEAST IMPORTANT.

	1 (Most important)	2	3	4	5	6 (Least important)
Minimized time to initial coverage	○	○	○	○	○	○
Minimized inter-individual efficacy variation	○	○	○	○	○	○
Minimized out-of-pocket cost	○	○	○	○	○	○
Minimized special handling	○	○	○	○	○	○
Maximized duration of vaccine response	○	○	○	○	○	○
Optimized opioid coverage	○	○	○	○	○	○

How OPPOSED OR SUPPORTIVE would you be regarding VOLUNTARY use of VBT for management of OUD and its associated risks IN ADULT PATIENTS under the circumstances below?

	Very Opposed	Somewhat Opposed	A Little Opposed	Neutral	A Little Supportive	Somewhat Supportive	Very Supportive
Drug court sentencing	○	○	○	○	○	○	○
Hospital discharge following overdose	○	○	○	○	○	○	○
In recovery from OUD and currently abstaining	○	○	○	○	○	○	○
Prophylactic use in at-risk individuals	○	○	○	○	○	○	○
Recent diagnosis of OUD with ongoing use	○	○	○	○	○	○	○

How OPPOSED OR SUPPORTIVE would you be regarding MANDATORY use of VBT for management of OUD and its associated risks IN ADULT PATIENTS under the circumstances below?

	Very Opposed	Somewhat Opposed	A Little Opposed	Neutral	A Little Supportive	Somewhat Supportive	Very Supportive
Drug court sentencing	○	○	○	○	○	○	○
Hospital discharge following overdose	○	○	○	○	○	○	○
In recovery from OUD and currently abstaining	○	○	○	○	○	○	○
Prophylactic use in at-risk individuals	○	○	○	○	○	○	○
Recent diagnosis of OUD with ongoing use	○	○	○	○	○	○	○

How LIKELY OR UNLIKELY would you be to SUPPORT the VOLUNTARY use of VBT for management of OUD and its associated risks IN THE PATIENT POPULATIONS BELOW?

	Very Unlikely	Somewhat Unlikely	A Little Unlikely	Neutral	A Little Likely	Somewhat Likely	Very Likely
Children	○	○	○	○	○	○	○
Pregnant Women	○	○	○	○	○	○	○
Prisoners	○	○	○	○	○	○	○

How LIKELY OR UNLIKELY would you be to SUPPORT the MANDATORY use of VBT for management of OUD and its associated risks IN THE PATIENT POPULATIONS BELOW?

	Very Unlikely	Somewhat Unlikely	A Little Unlikely	Neutral	A Little Likely	Somewhat Likely	Very Likely
Children	○	○	○	○	○	○	○
Pregnant Women	○	○	○	○	○	○	○
Prisoners	○	○	○	○	○	○	○

How SIGNIFICANT do you PERCEIVE the BARRIERS below to be when considering POTENTIAL implementation of VBT for management of OUD and its associated risks?

	Not at All Significant	Slightly Significant	Somewhat Significant	Very Significant	Extremely Significant
Insufficient time	○	○	○	○	○
Lack of provider availability	○	○	○	○	○
Lack of treatment affordability	○	○	○	○	○
Lack of treatment access	○	○	○	○	○
Patient refusal	○	○	○	○	○

How CONCERNING do you PERCEIVE the ETHICAL ISSUES below to be when considering POTENTIAL implementation of VBT for management of OUD and its associated risks?

	Not at all Concerning	Slightly Concerning	Somewhat Concerning	Very Concerning	Extremely Concerning
Confidentiality breaches	○	○	○	○	○
Access inequality	○	○	○	○	○
Promotion of risky behavior	○	○	○	○	○
Reduced patient autonomy	○	○	○	○	○
Inefficient use of scarce resources	○	○	○	○	○

RANK the following areas of consideration in regard to future implementation of VBT for OUD, from MOST to LEAST IMPORTANT for further study.

	1 (Most Important)	2	3 (Least Important)
Efficacy	○	○	○
Ethics	○	○	○
Logistics	○	○	○

How HELPFUL OR HARMFUL do you PERCEIVE the implementation of VBT for OUD would be in regard to patient care at your primary practice site?

- ○ Very Helpful
- ○ Somewhat Helpful
- ○ A Little Helpful
- ○ Neutral
- ○ A Little Harmful
- ○ Somewhat Harmful
- ○ Very Harmful

Thank you very much for taking the time to complete this survey!

D. APPENDIX C

Read the Following Text Prior to Beginning the Focus Group

Thank you very much for your interest in participation in this research study. Remember that participation is voluntary and you may leave the study at any time. This session will be recorded, but no personal identifying information will be collected. In order to preserve anonymity, please try to avoid using names during conversation. Although we do not anticipate collecting any stigmatizing or personally sensitive information, everything talked about during the focus group conversation should nevertheless be kept confidential. Please do not share your experiences outside of this room. Please ask any questions that you have at this time, after which we will begin the focus group.

1. **Opinions on opioid crisis**

- This section aims to gain student perspectives about the opioid crisis. What are the most pressing issues? What initiatives do they already know about? How confident are they in what they know?
    1. What do you know about the opioid epidemic?
        - What kinds of experiences have you had in practice, at your job, or on IPPEs?
    2. What can pharmacists do to combat the opioid epidemic?
    3. What are other health professions (MDs/PAs/nurses/social workers/psychiatrists) doing to combat the opioid epidemic?
    4. If you could change one factor of the opioid epidemic right now, what would you change?

2. **Patient demographics**

- In this section and coming sections, I hope to learn what stereotypes students may have about patients on chronic opioid therapy or medication-assisted treatment (MAT) and gauge for areas of discomfort/lack of confidence when consulting.
    1. What kinds of patients may be most susceptible to opioid overdose?
    2. What percentage of patients do you see are screened for excessive alcohol use?
    3. Roughly what percentage of patients you've seen are prescribed chronic opioid therapy?
        - What percentage of patients do you feel are at high risk for developing OUD?

3. **Medication Utilization (MAT specifically)**

- This section should build on the last. I hope to gauge what students know about MAT.
    1. If I say MAT, what do you interpret that to mean?
    2. What barriers exist for patients looking to get treatment?
    3. How important is a patient's adherence to MAT treatment regimens?
    4. What kinds of things can pharmacists do to minimize barriers to treatments?

4. **Opinions about current MATs**

- This portion of the focus group specifically gets at how well students feel that MAT works.

1. What are your opinions about the efficacy of MAT?
    - Does it work well?
2. What are the treatment goals for MAT?
3. How can MAT complicate treatment when opioids may be necessary (i.e. surgery, trauma, etc.)?
4. What do you know about Vivitrol?
5. How do you think efficacies compare among the different types of MAT available?

5. How are you implementing MAT?

- Next, I will ask students about current systems barriers in place for MAT.
    1. Physicians must have a specific DEA license and training to prescribe medications for MAT. Do you think pharmacists should also have specific license requirements? Why or why not?
    2. What kinds of barriers keep pharmacists from implementing MAT?
    3. What can pharmacists do to address the patient-level barriers you've already identified?

6. Opinions on Behavioral interventions

- The next item of discussion focuses on what students have learned about behavioral and mindfulness therapy as well as their opinions on the efficacy of this intervention.
    1. What do you know about behavioral therapy for substance use disorders?
    2. How would you characterize talk therapy (positive/negative)?
    3. What connections are there between CBT and motivational interviewing?
    4. How can pharmacists work to coordinate care for patients using eclectic forms of treatment (i.e. therapy with medication)?

7. How can you implement behavioral interventions in your practice?

- This next section aims to address potential interprofessional relationships that can enhance care for patients with OUD or other mental health conditions.
    1. What kinds of interprofessional interactions might occur between pharmacists and psychiatrists?
        - How can care be streamlined from therapy to pharmacy?

8. Awareness of vaccine-based therapy

- In this section, I will simply ask what students already know about vaccine-based therapies for OUD/other substance use disorders.
    1. What do you know or what have you heard about vaccine-based therapies for opioid use disorder or other substance use disorders?

9. Opinions about vaccine-based therapy

- Next, I will briefly describe the work being done about vaccine-based therapy. The following is a draft of what I plan to include in the survey tools:

- Recently, there have been new advances in vaccine science, especially in vaccines used to treat substance use disorders. One vaccine in the preclinical stage aims to program the body to create antibodies to heroin and other types of opioids. When someone who has been vaccinated uses an opioid, the antibodies will selectively bind to that target and conglomerate, thus preventing the drug to enter the CNS and stimulate the reward pathway.
    1. In general, what are your thoughts about this therapeutic approach?
    2. What concerns might you have?
    3. What are some pros and cons of this idea?
        - Similar vaccines for cocaine and nicotine have already been developed and are undergoing clinical trials in humans.

10. How might stakeholders implement VBT?

- This portion aims to get students thinking about different barriers to bringing VBT to clinical practice.
    1. What resistance might there be to bringing an anti-opioid vaccine to market?
        - Think about various groups of people: patients/families/parents, doctors/prescribers, insurance companies/payers, and the general public.
